# Software-Defined Networking: Categories, Analysis, and Future Directions

**DOI:** 10.3390/s22155551

**Published:** 2022-07-25

**Authors:** Mudassar Hussain, Nadir Shah, Rashid Amin, Sultan S. Alshamrani, Aziz Alotaibi, Syed Mohsan Raza

**Affiliations:** 1 Department of Computer Science, University of Wah, Wah Cantt P.O. Box 47010, Pakistan; 2 Department of Computer Science, COMSATS University Islamabad, Wah Campus, Wah Cantt P.O. Box 47010, Pakistan; nadirshah82@gmail.com (N.S.); smohsanraza@gmail.com (S.M.R.); 3 Department of Computer Science, University of Chakwal, Chakwal P.O. Box 48800, Pakistan; 4 Department of Information Technology, College of Computer and Information Technology, Taif University, Taif P.O. Box 21944, Saudi Arabia; susamash@tu.edu.sa; 5 Department of Computer Science, College of Computers and Information Technology, Taif University, Taif P.O. Box 21944, Saudi Arabia; azotaibi@tu.edu.sa

**Keywords:** SDN, network testing and verification, flow rule installation mechanisms, network security and management, memory management, SDN emulators and simulators, SDN programming languages, SDN controller platforms

## Abstract

Software-defined networking (SDN) is an innovative network architecture that splits the control and management planes from the data plane. It helps in simplifying network manageability and programmability, along with several other benefits. Due to the programmability features, SDN is gaining popularity in both academia and industry. However, this emerging paradigm has been facing diverse kinds of challenges during the SDN implementation process and with respect to adoption of existing technologies. This paper evaluates several existing approaches in SDN and compares and analyzes the findings. The paper is organized into seven categories, namely network testing and verification, flow rule installation mechanisms, network security and management issues related to SDN implementation, memory management studies, SDN simulators and emulators, SDN programming languages, and SDN controller platforms. Each category has significance in the implementation of SDN networks. During the implementation process, network testing and verification is very important to avoid packet violations and network inefficiencies. Similarly, consistent flow rule installation, especially in the case of policy change at the controller, needs to be carefully implemented. Effective network security and memory management, at both the network control and data planes, play a vital role in SDN. Furthermore, SDN simulation tools, controller platforms, and programming languages help academia and industry to implement and test their developed network applications. We also compare the existing SDN studies in detail in terms of classification and discuss their benefits and limitations. Finally, future research guidelines are provided, and the paper is concluded.

## 1. Introduction

Traditional networks are complex and hard to manage because all the functionalities of the data, control, and management planes are vertically and tightly coupled in forwarding devices [1]. In traditional networks, the control plane, with the help of routing protocols, forwards data packets as per network policies. Due to this vertical integration and the tightly coupled nature of forwarding devices, network management becomes difficult, and performance tuning becomes challenging. Moreover, the network applications and services of the current information age have become more complex and demanding, so it is necessary that the Internet be able to evolve to address these new challenges. To resolve such issues, the idea of “programmable networks” has been proposed to facilitate network evolution. In this regard, two concepts, active networking [2] and programmable networks [3], were explored. Active networking refers to network intelligence (as opposed to typical packet processing), where network nodes have the capability of performing customized operations on packets. Programmable networks permit the controlling of network nodes’ behavior and flow control through software. Later, the 4D project [4,5,6] proposed a clean slate design that is based on four planes: decision, dissemination, discovery, and data. It emphasizes the separation of routing decision logic and protocols governing the interaction between forwarding devices. The decision plane has a network-wide view of the network topology and installs configuration commands at the data plane for communication. The dissemination and discovery planes provides efficient communication between the decision and data planes. Ethane [7] proposed a new network architecture for enterprises that allows network managers to configure and control the whole network by using a centralized controller. These research works proposed a clear foundation for separation between the data and control planes, which resulted in the introduction of software-defined networking (SDN). SDN provides a real-world implementation of a suite of networking software that allows a network to be centrally controlled. It is not the first and only solution that accepts separation and programmability. However, it has wide acceptance in both academia and industry due to the rapid innovation in both the control and data planes. A group of network operators, service providers, and vendors have created the Open Network Foundation (ONF) [8], which is an industrial-driven organization to promote SDN and standardize the OpenFlow Protocol (OFP) [9]. On the academia side, the OpenFlow Network Research Center [10] has been created, with a focus on SDN research.

SDN [11,12,13,14,15] is an emerging form of network that resolves these limitations by separating network control and management from the data plane to reduce complexity and increase network management. This separation of the control and data planes leaves network switches as simple forwarding devices. However, the network control is shifted to the centralized logical entity called the controller, which acts as the network’s brain and maintains a global view of the network and programming abstractions. It offers a programmatic control of the entire network and provides real-time control of underlying devices to network operators. The management plane specifies network applications, such as network policies, network monitoring, load balancing, and so on, which are implemented by the network administrator based on the application environment and the user’s requirements. In this way, network management turns out to be simple, which reduces network rigidness. All three of the SDN planes interact with each other by using application programming interfaces.

### 1.1. Application Programming Interfaces

**Figure 1:**
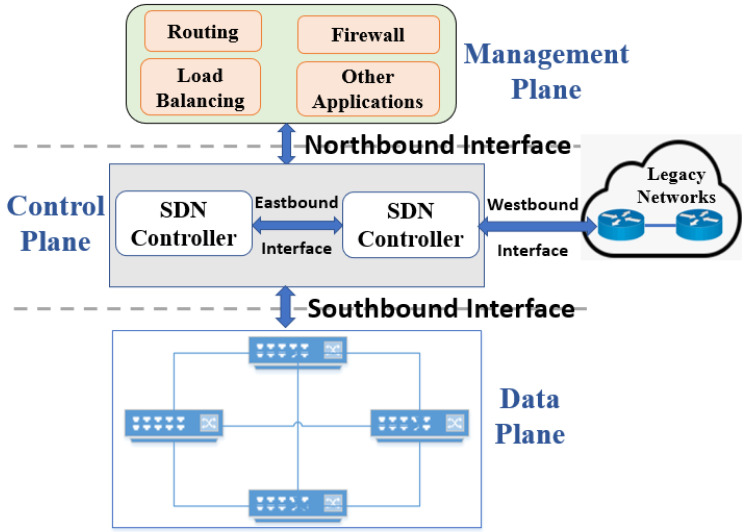
SDN system architecture.

Application programming interfaces (APIs) [16] are very important in SDN; they provide communication between the data, control and management planes. The well-known APIs are southbound APIs (SBI), northbound APIs (NBI), and in the case of distributed controllers, east/westbound APIs. These APIs are architectural components of SDN, and they are used to configure forwarding devices or network applications. The SDN layered architecture, including APIs, is shown in Figure 1.

**Figure 2:**
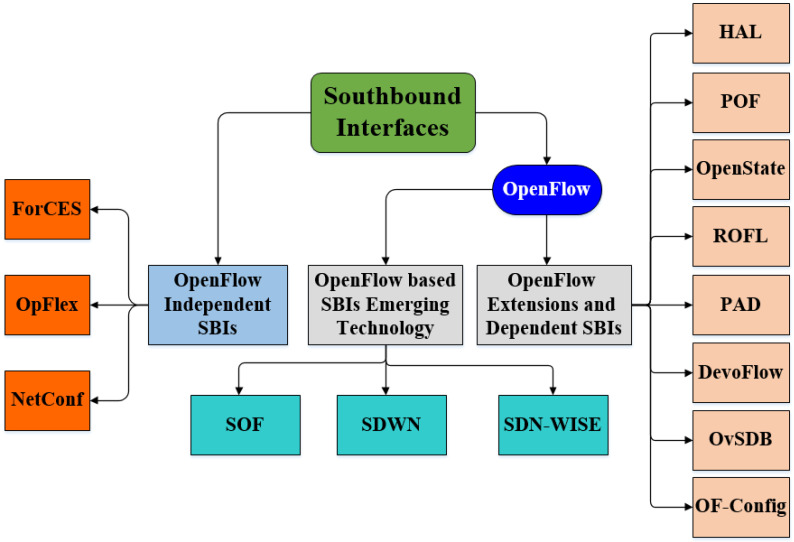
Southbound API proposals.

1. Southbound API: This is an SDN enabler that provides a communication protocol between the control plane and data plane. It is used to push configuration information and install flow rules at the data plane. It also provides an abstraction of the network device’s functionality to the control plane. Moreover, it allows the discovery of network topology, defines network flows, and implements requests sent by the management plane. SBI is critical with respect to its availability as well as secure communication. In absence of any one of these parameters, it may result in the malfunctioning of forwarding devices. It faces challenges of heterogeneity, vendor-specific forwarding devices, and programming languages. OFP is commonly used as an SBI that provides secure channel between the control and data plane to install flow rules. SBI proposals are categorized based on whether they are OpenFlow-dependent, OpenFlow-independent, or emerging technology, all of which are shown in Figure 2.
  - OpenFlow-Dependent SBI Proposals: These include OpenFlow-based SBI proposals with the addition of new features or in its newer versions. DevoFlow [17] modifies the OpenFlow model to permit network operators to focus on flow rules, which are essential for network management, by breaking the coupling between network control and global visibility. It helps to reduce internal communication between the control and data planes. The Revised OpenFlow Library (ROFL) [18] provides an API that offers much better usability by hiding details of OFP versions to make application development easier. It utilizes the extensible datapath daemon (xDPd) framework, which facilitates creating SDN data path elements. The hardware abstraction layer (HAL) [19] separates hardware-specific control and management functionalities from the forwarding devices in order to make legacy network nodes such as OpenFlow compliant. It results in decreasing the complexity of network devices, and the problem of vendor-specific features is resolved. OpenState [20] states that a central controller should not be provided with full control. It also proposes that the programmers can implement states in the forwarding devices instead of the central controller. Protocol-oblivious forwarding (POF) [21] offers a reactive mechanism that requires forwarding devices to extract keys and process packets by using packet headers, which leads to overhead. The programming abstraction datapath (PAD) [22] reveals the programmability of switch capabilities and offers SBI for optical switches. The open virtual switch database (OvSDB) [23] and OpenFlow configuration protocol (OF-Config) [24] build an association between the control and data planes and facilitate configurations in OpenFlow.
  - OpenFlow-Independent SBI Proposals: P4 runtime SBI [25] helps to solve the problem of OFP. It is an open, extensible, customizable platform that offers a new way for the control plane to control forwarding devices and solves the limitations of OFP. Forwarding and control element separation (ForCES) [26] aimed to replace OpenFlow. It enables the separation of control and forwarding elements that are in the same physical device without modifying the traditional networking architecture and without involvement of an external controller using a logical function block. OpFlex [27] supports communication between the controller and data plane; however, its provision of service is different in comparison with OpenFlow. It resolves the scalability problem by distributing load to the forwarding devices. NetConf [28] uses a remote procedure call paradigm to manage and configure network devices remotely. It was already present before the emergence of SDN and offers a very simple API to send and receive full or partial configuration datasets.
  - OpenFlow-Based SBIs Emerging Technology: Sensor OpenFlow (SOF) [29] is upgraded as per the specifications of low-capacity sensor nodes. It is based on OpenFlow and aimed to address the challenges of flow and congestion control. It offers the ability to redefine flow tables as per the specific addressing schemes of wireless sensor networks (WSN) to install flow rules on sensor network devices. Software-defined wireless networks (SDWN) [30] aimed to decrease energy consumption in WSNs with the help of duty cycles and in-network data aggregation. Duty energy minimizes consumption by turning radio off in case of idle periods, and in-network data aggregation is also helpful in this regard. Its protocol architecture utilizes both generic and sink nodes. In addition, it supports elastic flow rules due to its nature, unlike traditional OpenFlow. SDN for wireless sensors (SDN WISE) [31], implemented in OMNet++, aimed at reducing sensor nodes’ communication with the controller, in addition to making sensor nodes programmable. SOF and SDWN utilize a central controller to provide theoretical details; however, SDN-WISE is based on a distributed controller paradigm, using an ONOS controller [32] to provide services based on practical implementations.
2. Northbound API: Control and management planes use NBI to provide programmability to application developers. NBI is very important with respect to the ability of SDN adoption to support variety of SDN applications. A wide range of NBIs are offered by current controllers and programming languages due to the lack of standardization. In addition, some programmers and many controller platforms use the REST API as NBI. One of the initiatives of ONF is the Northbound Interface Work Group (NBIWG) [33], which was formed with the intention of standardizing NBI.
3. East/Westbound API: Inter-controller communication of SDN domains is established using eastbound API. Westbound API is responsible for communication from the legacy domain to SDN domain. Central network control is the key feature of SDN; however, the single controller can handle only a limited number of forwarding devices. To accommodate the exponential increase in forwarding devices and for large-scale networks, distributed controllers have become a requirement. Eastbound APIs are used to import/export information among distributed controllers [34,35,36], and westbound APIs enable communication between legacy network devices and the controllers [37,38,39].

### 1.2. Network Configuration and Flow Rules Installation

Computer networks are mainly configured based on access control list (ACL) policies and routing protocols. The ACL policies are the set of rules that instruct network devices to function as per the requirements of users, applications, and/or organizations. The routing protocols help to find best path between source and destination. In SDN, the ACL policies are configured at the network control plane, and based on those policies, flow rules are generated and installed at forwarding devices. These policies often change in computer networks as per the demands of hosts or changes in network topology to allow or deny specific communication [40]. The SDN programming languages (for example, Pyretic [41], Frenetic [42], and Maple [43]) help to specify ACL policies as per the application environment via parallel and sequential composition operators for efficient implementation of policies. Whenever a host initiates a communication process, the forwarding device (switch) checks flow rules for that communication in its flow table. If a flow rule does not exist, then it sends a digest packet to the controller. The controller calculates the best path between the source and destination host according to the network topology and ACL policy. The flow rules are installed along the computed best path, and based on these flow rules, communication takes place. The switch stores these flow rules in its flow table until the timeout value expires due to inactivity. There are two types of timeout values. The first type is soft timeout, which states that the flow rule is deleted from the switch flow table if it is not used for a defined number of seconds. The second type is hard timeout value, which states that the flow rule is deleted a after certain number of seconds [44]. These timeout values depend on the application environment and controller platform [45].

The flow rules are installed based on reactive, proactive, and hybrid mechanisms [46]. In a reactive mechanism, when a packet is received at the switch, it looks up its flow table to initiate the forwarding process. In the case of flow rule matching, the respective packet is forwarded as per flow entry. However, in the case of non-matching, it sends a digest packet to the controller, which reacts by consulting the network topology, routing protocols, and ACL policies. It computes and installs the flow rule along the path between the source and destination via packet-out messages. All subsequent packets follow the same path without intervention from the controller. In this approach, only required flow rules are installed as per the request from communication hosts, so it helps to reduce the load in the flow tables of the data plane. This, in turn, efficiently utilizes ternary content-addressable memory (TCAM) resources. In a proactive approach, the flow rules are pre-populated, that is, populated before the first packet of a flow arrives at a switch based on network policies, routing protocols, and network topology [32]. This approach reduces the flow rules setup delay as well as the number of signaling messages due to the predefined actions and their flow rules installation before the arrival of packets. Therefore, the packets are forwarded just by matching flow rules, which saves a large amount of time. However, the TCAM resources of switches are not efficiently utilized due to the installation of flow rules for which communication is not desired. The hybrid approach is combination of reactive and proactive and is flexible in the sense that it includes the best characteristics of both proactive and reactive approaches. It offers flexibility and robustness and helps to reduce communication delays.

### 1.3. SDN Advantages

SDN has many advantages over traditional networks owing to the lower maintenance, ease of management, and implementation of ACL policies [47,48]. It simplifies network management and control by managing the whole network from the centralized controller. Moreover, forwarding devices (switches) become simplified as network intelligence is shifted to the controller; thus, these devices are left with very basic functionalities as they only need to act according to the instructions from the controller and do not require understanding and processing heterogeneous algorithms and protocols. In addition, the forwarding devices also help the controller for route computations and link/node monitoring, along with other tasks such as network management and diagnostics [49]. SDN has numerous advantages compared to traditional networking. Some of them are defined below:

- SDN Centralized Management and Control: SDN’s centralized management and control of networking devices helps to reduce complexity.
- Directly Programmable: The network control plane can be directly programmable as it is separated from the data and management planes.
- Easier Network Management and Automation: It offers easier network management and automation via common APIs to program the applications due to the provision of abstractions by the controller platform.
- Rapid Innovation: It allows rapid innovation, as there is no need to configure each device and no need to wait for new releases from vendors.
- Programmability: The network is programmable with the help of network applications that are installed at the control plane to offer vendor independence.
- Flexibility: It provides a flexible network architecture that protects existing investments while future-proofing the network.
- Flow-Rules-Based Forwarding: Forwarding decisions are based on flow rules (instead of destination-based addresses), which broadly implement flow rule matching and action criteria.
- ACL Policy Implementation: It allows network administrators to apply ACL policies at a more granular level in a highly abstracted automated fashion.
- Usability: It provides better user experience by centralizing network control and making state information available to higher-level applications as it can adopt dynamic user needs easily [50,51,52,53].
- Security: It provides centralized security control, which improves network visibility through security management. In addition, it offers robust control over network infrastructure to develop efficient and effective security mechanisms [54,55] to detect and prevent security attacks [56,57].
- On-Demand Quality of Service: It utilizes the SDN central control intelligence for the aggregation of services from long-term evaluation (LTE) and the wide area network (WAN) to tackle the increasing computational demands of mobile users [58].
- Traffic and Resource Categorization in Edge Network: The collaboration of network function virtualization (NFV) and SDN in virtualized network infrastructures (multiple NFV, virtual network function (VNF), service function chaining) provides a customized QoS for residential network requests via differentiated treatment of each clustered and tagged piece of traffic in the edge network. Traffic encompasses audio and video streaming. SDN assists in the realization of differentiated behavior of underlying hardware network resources [59].
- Mobility Support for Internet of Vehicles (IOV): SDN enables intelligent remote clouds for the computation of tasks offloaded by speedy vehicle to the roadside unit (RSU). The controller supports the RSU for implementing the communication path between the RSU and fog node with adequate resources in a predictive fashion. Consequently, the fog node capabilities log at the controller tends to the optimal computation of IOV jobs by fog nodes [60].
- Topology Discovery: SDN has centralized services for event-based topology discovery. Tree exploration discovery protocol (TEDP) outperforms the OpenFlow protocol and standard link layer discovery protocol (LLDP) in terms of the reduction of extra packets for topology discovery. The tree exploration discovery protocol (TEDP) leverages the topology graph mapping by using the probe packets that are responsible for signaling the SDN controller to a single forwarding device. As a result, the discovery mechanism is optimized in SDN compared to LLDP, which introduced load in IP networks [61].
- Load Balancing Support in Future Networking: Research work comprises enabling the SDN infrastructure among the multiple NFV nodes and service function chaining aiming to reduce the delay of state migration of VNF. Concretely, the objectives are to cope with the limited resource capabilities of NFV nodes, meeting the desired QoS by infrastructure, and ensuring the least end-to-end delay for computational states migration by confining the OpenFlow-enabled devices’ capabilities in 5G core networks [62].
- Fault Localization: In SDN, centralized management is used for localizing the failure and reconfiguration help to localize faults. It handles failures in a proactive fashion based on the prediction of service unavailability [63].
- Programmable Reachability Optimization: SDN handles firewall problems in term of conflicting rules automatically. Similar research works offer improvements with the least computational overhead in reachability optimization and conflict debugging problems [64].
- Support for Cellular 6G Network: Future networks such as 6G require SDN-enabled softwarization and management for remote and machine learning application decision-aware re-configurations in network resources [65,66,67].
- Security-Aware Communication in Future Autonomous Networks: Softwarized policy implementation architecture can enhance the security among autonomous systems that have the least human interactions, and consequently, it can mitigate the security risk of inter-domain communication [68,69].

### 1.4. Organization

**Figure 3:**
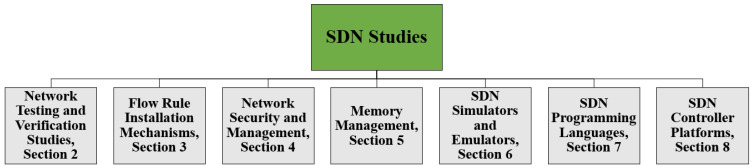
SDN studies categorization hierarchy comprised of seven sections (Section 2, Section 3, Section 4, Section 5, Section 6, Section 7 and Section 8).

The rest of paper is organized as follows. Section 2 compares network testing and verification studies, which look at mechanisms for testing and debugging techniques. Section 3 includes flow rule installation mechanisms that comprise reactive, proactive, and hybrid flow rule installation mechanisms. In Section 4, network security and management issues related to SDN implementation in different scenarios are discussed, along with solutions to the problems. Section 5 comprises memory management studies, which help to utilize precious TCAM resources in an efficient way. Section 6 includes emulators and simulators for SDN that help to implement, test, and simulate research problems. Section 7 comprises SDN programming languages, which facilitate programmers to develop network applications for effective communication. Section 8 consists of SDN controllers that provide a platform to control the data plane by installing flow rules via southbound APIs and network applications via northbound APIs. The categorization hierarchy is shown in Figure 3. Section 9 discusses existing SDN studies. Finally, Section 10 includes the future research guidelines, and Section 11 concludes this paper.

## 2. Network Testing and Verification

NDB [70] is a network debugging tool to debug SDN via breakpoints, watches, and packet backtraces. It works like GNU debugger (GDB) [71], which pauses execution at a breakpoint and shows the sequence of events that led to that breakpoint. Proxy and collector are its two major components. The proxy creates a postcard message received from the data plane and sends it to the control plane. On receiving this message, the collector saves postcards and produces backtrace for the listed data packets. Finally, by using the hash table data structure, the collector keeps the postcards from where these can be recovered effectively.

Veriflow [72] detects network-wide invariants in real time and generates alerts or blocks the occurrence of events. On generation of flow rules from the controller, these flow rules are sent to VeriFlow for checking the network-wide invariants. It generates a notification on detection of network-wide invariants for the network admin or the flow rules are blocked. Otherwise, the flow rules are sent to the data plane. It verifies the flow rules for network-wide invariants in the following three steps. First, the network is segmented in a collection of equivalence classes (ECs) by using network routing policies. Secondly, VeriFlow creates individual graphs for the specific equivalence class that denotes the respective network behavior. Thirdly, with the help of these graphs, the status of the network invariant is identified. It stores network information, for example, ACL policies, in trie data structures [73] and computes ECs in a systematic way. In addition, other research works [74,75,76] have also resolved issues of debugging and testing in traditional as well as SDN networks. These can detect network anomalies, ensure data plane consistency [77,78], and remove conflicts of different network applications to execute in a parallel manner [79].

In [80], the problem of flow rule installation from controller to switches is addressed. Due to this problem, the packets may deviate from their intended paths, which results in access control violations. The rule enforcement verification (REV) mechanism enables the controller to ensure the correct installation of flow rules along the correct path at the switches. It proposes a compressive message authentication code (MAC) to compress switch-to-controller communication traffic, which reduces a significant amount of bandwidth cost. Finally, it presents a heuristic flow selection algorithm, which allows the controller to verify many fewer flows for rule coverage. This results in avoiding adversity to the temper flow rule installation, thereby ensuring proper implementation of access control. NICE [81] detects network-wide invariants via model checking and symbolic execution. To detect network-wide invariants, NICE generates a stream of packets under various conditions to test SDN. In SDN, the details of topology, including switches and hosts, are available at the controller. After that, the space of possible system behavior and network-invariant conditions are tested. The required search strategy can also be configured by the programmer. NICE provides an output of instances of network invariants. Additionally, it provides traces of the inevitable consequence of property violations to reproduce them.

FPB [82] offers an efficient buffer management scheme at the data plane to avoid packet disorder and minimize the packet drop ratio by forwarding only the first packet of a flow to the controller while subsequent packets are buffered. HSA [83] is beneficial for system admins, as they can statistically examine their networks for invariants, for instance, network violations, black holes, loops, traffic isolations, and so on. HSA has the ability to check various hosts, network traffic, and the isolation of users. For example, it can provide details regarding questions such as “Can host A be prevented from talking to host B?” and so on. In this tool, a geometric approach is opted for as generalization for packet classification. PyResonance [84] utilizes the Pyretic language to implement state-based network policies. It uses Pyretic composition operators to express these policies and to compose multiple tasks by determining the state of their forwarding behavior with the help of a finite state machine. In this way, multiple independent states can be defined along with their forwarding behavior to handle the state change of multiple events.

PGA [85] provides automatic and conflict-free policies, for example, network policies, load balancing [86], and so on. It examines various network policies that are individually stated for any conflict. In different situations, network policies conflict with each other due to various perspectives. The graph composition is very helpful to express conflict-free network policies. As a next step, these policies are forwarded to a graph composer through a PGA user interface (UI). It resolves conflicts or gives some possible suggestions to the network admin in this regard. Finally, it generates error-free/conflict-free graphs. A service function chain-based approach to specify and verify ACL policies is presented in [87] to detect anomalies in ACL policies prior to deployment. In order to achieve the desired goal, the forwarding policies are formally represented, and a set of anomalies are detected against the set of flow rules for the respective policies. In addition, it also provides a provision for network administrators to specify their own anomalies. The results state that the proposed approach can verify anomalies of a reasonably sized network in milliseconds. Moreover, the research works in [75,76] are useful to troubleshoot, debug, and detect anomalies in communication networks.

### Summary and Lessons Learned

The summary of network testing and verification studies are presented in Table 1, and the following conclusions are drawn based on these studies. NDB [70] helps to debug SDN networks via breakpoints and packet backtraces. These primitives help in locating the order of events that led to error conditions. Though NDB can identify error conditions, it does not fix the error conditions. VeriFlow [72] checks network invariants in real time and generates an alarm or blocks these events from occurring. It checks network invariants in real time. However, it does not work in multi-controller architecture, and its verification process does not support delay-sensitive applications in which flow rule installation is continuously in flux, which is most likely desired for the least delay in forwarding devices. NICE [81] uses model checking and symbolic execution to detect bugs and invariant conditions in SDN applications. However, it is unable to test a controller implemented in the same language. FPB [82] provides an efficient buffer management mechanism at the switch level to avoid per-flow packet disorder, which helps to minimize the packet drop ratio. HSA [83] facilitates network administrators to statistically analyze their networks for network-wide invariants. However, it only works for static networks and lacks the ability to detect network policy change with dynamic change in ACL policies.

PyResonance [84] utilizes the composition operator of the Pyretic language to implement state-based network policies to predict a possible network’s forwarding behaviour. PGA [85] provides automatic and conflict-free policies by examining various network policies that are individually stated for any conflict. In PGA, the focus is on implementation of various policies in such a way that conflict does not occur. The research works [75,76] debug traditional as well as SDN environments to detect network anomalies, ensure consistency of the data forwarding plane [77,78], and allow several applications to run in parallel in a non-conflicting way [79]. However, all these mechanisms lack the ability to detect network policy change and delete conflicting flow rules along with installation of new flow rules as per new network policies to avoid packet violations. There is still a need to develop tools that can detect bugs in real time, along with the mechanisms to correct those bugs. This will help to avoid network inconsistencies, which will result in increasing network efficiency and QoS.

## 3. Flow Rule Installation Mechanisms

ORPP [88,89] provides two flow rule placement frameworks: OFFICER and aOFFICER. It helps to define and install flow rules at the data plane by following all technical and non-technical requirements. The first framework, called OFFICER, helps to define and install flow rules for the set of known requirements at a specific time internal. The second framework, aOFFICER, helps to compute and install flow rules for unknown sets of requirements, which vary over a specific time interval. Both these frameworks are quite useful and effective to place flow rules at the data plane. vCRIB [90] provides a mechanism that proposes an abstraction for specifying and managing flow rules for network operators at data center networks. In addition, in order to achieve better performance and resolve scalability issues, it helps to partition and install flow rules at hypervisors and switches. DevoFlow [17] presents a model to modify the OpenFlow model that permits network operators to focus on flow rules, which are essential for network management, by breaking the coupling between network control and global visibility. This mechanism helps to reduce internal communication between the control and data planes. Firstly, it minimizes the need to transfer statistics for tedious flows. Secondly, it minimizes the need to invoke the control plane for most flow setups. This helps to maintain a certain level of visibility by minimizing communication overhead between the control and data planes. However, the prototype is not simulated on actual packets.

Infinite CacheFlow [91] proposes a hybrid switch design that depends on flow rule caching to increase the flow rule tables space of switches at quite a low cost. However, it may result in more packet violations for flow rules that are stored at the data plane if corresponding ACL policies change at the controller. SwitchReduce [92] proposes a technique with the assumption that the number of flow match rules at any switch should not exceed the set of unique processing actions to decrease the switch state and controller involvement in SDN. The proposed approach can reduce flow entries up to 49% on first hop switches, and up to 99.9% on interior switches. In addition to that, flow counters are also reduced by 75% on average. It shows some failures due to topology changes. However, analysis of packet violations in case of change in policy is not performed on larger data centers. In [93], a cache algorithm strategy to store flow rules at switches called “least recently used" (LRU) is proposed, which reduces communication overhead between the control and data planes. This technique helps to avoid the cache-miss problem by keeping recently used flow entries in the switch flow table, which increases the flow entry matching ratio. This approach also ignores the case when network flow rules change for the flow entries present at the data plane.

In [94], a flow rule multiplexing approach is proposed that optimizes both the flow rule allocation as well as the traffic engineering. It works by installing identical set of flow rules at different calculated paths for a whole session instead of installing at each switch. It is tested via the ITALYNET network topology, and results reveal that the proposed mechanism saves TCAM resources and guarantees high QoS satisfaction. DomainFlow [95] presents a flow level control and granularity-based mechanism in ethernet switches by using the OpenFlow protocol. This research utilizes exact matching and network slicing to enable practical flow management. It only supports a limited number of flows with commodity switches and cannot be implemented with a large number of flows. SourceFlow [96] presents a mechanism that can handle many flows without affecting flow granularity, in addition to minimizing the problem of costly and power-consuming search engine devices from the core nodes. Moreover, it facilitates growing networks without compromising scalability. In [97], an SDN-based proactive flow rules installation mechanism is proposed for efficient communication in the Internet of things (IoT). It resolves the problem of flow installation delay as well as congestion due to packet-in messages, which save energy and other potential resources of network nodes.

In SDN, flow rules are installed at the data plane based on exact matching [98] or wildcard-based matching [99]. The wildcard-based matching improves the reusability of flow rules and reduces packet-in messages. It improves scalability at both the data and control planes. However, in the case of exact matching, almost every flow passing the switch will generate a packet-in message to the controller, which exhausts precious resources. To resolve this problem, some researchers suggest using a load-balancing mechanism by installing proactive flow rules on multiple switches [100] or reactive caching flow rules in each switch. In [101], an SDN-based wildcard rule caching mechanism, namely caching in buckets (CAB), is proposed by partitioning the field space into buckets and caching those buckets along with respective flow rules for efficient flow rules placement. This mechanism resolves the flow rule dependency problem with much less overhead, along with reducing flow setup time and saving network bandwidth and flow setup requests. In SDN, most of the operations are performed at the central controller, such as network topology management, flow rules installation, load balancing, and so on. These tasks sometimes overburden the controller, which becomes unavailable for some required operations. DIFANE [102] is a solution to this problem in which most of the functionality is placed on the network switches. For this purpose, the controller delegates the flow rules to some of the network switches that are called authority switches. These switches give the flow rules to the other switches for a specific path. In this way, entire-network communication becomes possible.

Mobi-Flow [103] presents a system architecture for the movement of mobile nodes in SDN by using two components: path estimator and flow manager. The path estimator helps to find the possible positions of end users in the network based on the location history of the node. For this purpose, we keep track of the previous positions of the nodes in the network in the database. By using the order-k Markov prediction method, the next possible position of end users is predicted. If we have possible location information of the end user, then the flow manager determines the set of access points in the path for communication between source to destination. In [104], a novel technique is proposed to install flow rules at the SDN data plane before reaching packets at network devices. In SDN, it sometimes happens that subsequent data packets arrive at the switches where flow rules are not found, causing the discarding of packets. To solve this issue, a new technique is proposed that computes packet arrival delay and flow rule installation time. After this computation, if there is a delay between flow rule installation and packet arrival, then some delay is introduced to the packet at the predecessor switch.

In the current research work, in order to improve the flexibility and scalability of the entire network, a novel mechanism is proposed in which network policies are deployed on the network devices in a wildcard format. Only the most important policies are cached in the flow table, while unnecessary policies are removed as soon as possible. By using this mechanism, the risk of flow table overflow is reduced, and it also simplifies the network policy enforcement. The wildcard used in this technique requires a standard way of being adopted in the entire network. To cope with this problem, a network-wide wildcard rule engine is introduced for SDN that is known as BigMac [105]. BigMac works by advertising a layered model to publish the higher-level network policies. The policy model consists of a big switch abstraction and a logical network plane that specifies the different forwarding and management policies. When a new flow needs to be installed, the policy caching and mapping engine of BigMac is accessed to install flow rules on the entire path. Similarly, when scheduled traffic needs to be forwarded, BigMac deploys the requested flow rules on the entire path. The research works in [106,107] proposed mechanisms to effectively install flow rules at the data plane in case of policy change without packet violations due to old installed flow rules as per old policies. To implement these mechanisms, the generated flow rules as per policies are cached at the controller. In case of policy change, the proposed mechanisms detect this change and compute the shortest path to install the computed flow rules, in addition to deleting the old flow rules. ROCA [108] proposes a novel mechanism to detect and resolve network conflicts along with policy overlapping for effective communication in SDN. The proposed approaches help to resolve network policy conflicts and efficiently install flow rules at the data plane.

### Summary and Lessons Learned

The summary of flow installation mechanisms is presented in Table 2, and we draw the following conclusions based on these studies. ORPP [88,89] resolves the problem of the placement of flow rules by using two frameworks: OFFICER and aOFFICER. Both frameworks provide efficient mechanisms to install flow rules. vCRIB [90] provides abstraction to define and manage flow rules for data center operators by portioning and placing flow rules on switches and hypervisors. DevoFlow [17] helps to amend the OpenFlow protocol by allowing network operators to focus on only selected flows for network management to reduce overhead between the control and data planes. Infinite CacheFlow [91] solves the issue of the limitation of flow rules at switches due to the limited TCAM resources by proposing a hybrid switch design (hardware and software). SwitchReduce [92] minimizes the switch state and controller involvement in SDN. The mechanism in [93] reduces the communication overhead between controller and switches by storing flow rules at the data plane. In [94], a flow-rule-based multiplexing approach is proposed, which optimizes both the flow rule allocation as well as the traffic engineering. DomainFlow [66] presents a flow-level control- and granularity-based mechanism in ethernet switches by using the OpenFlow protocol. SourceFlow [96] presents a mechanism that can handle many flows without affecting the flow granularity, in addition to minimizing the problem of costly and power-consuming search engine devices from the core nodes.

In [97], a proactive flow rules installation approach is proposed for efficient communication in the Internet of things (IoT), which resolves the problem of flow installation delay and congestion due to packet-in messages. DIFANE [102] proposes a mechanism of relaxing the control plane by sharing control plane tasks with network switches. For this purpose, the controller delegates the flow rules to some of the network switches that are called authority switches. These switches give the flow rules to the other switches for a specific path. In this way, entire-network communication becomes possible. All these mechanisms help in installing efficient flow rules at the data plane, better utilizing precious TCAM resources, reducing communication overhead between control and management planes, and minimizing the communication load on the controller. These approaches help to install and manage flow rules at the data plane, reducing the load on the controller and effectively utilizing TCAM resources in SDN. There is still a need to investigate flow rule installations by utilizing proactive, reactive, and hybrid mechanisms in the case of ACL policy change to avoid maximum packet violations.

## 4. Network Security and Management

In this section, we discuss network security and management strategies in SDN. In [110], a token-based authentication mechanism is proposed that guarantees exclusive access of network resources to a certain flow for which the user/app has made the reservation. The proposed SDN-based system automatically reserves the resources of the users/apps for certain flows and creates a strong binding between them. Moreover, it resolves the reservation problem of dedicated access to specific resources in distributed environments and high-speed networks. In [111], the problem of network verification of middleboxes (for example, caches and firewalls) is proposed by checking all possibilities to verify the network reachability properties as per ACL policies. It works by slicing complex networks into small networks according to the correctness properties of network-wide verifications. In [112], a troubleshooting workflow is presented that is comprised of two phases. In the first phase, a binary search through the control stack is conducted to check for the occurrence of mistranslation. In the second phase, the scope of those elements that are responsible for the invariant violation is reduced. In this way, it makes it easy to identify the root causes of bugs, which helps network admins to troubleshoot their networks in an effective manner.

In [113], a priority-based flow rules security problem is highlighted, and a solution to the identified problem is presented. The problem is that the low-priority malicious flow rules can manipulate the whole OpenFlow network by making the high-priority flow rules fail. This, in turn, affects the whole data communication process. To solve the identified problem, the authors proposed a solution that is called switch-based rules verification (SRV). It works by leveraging the SDN controller to obtain the overall network view of the whole topology and detect the malicious flow rules. On detection of a malicious flow rule, the SRV module forwards warning messages and refuses the identified flow rule instantly. This solution helps to detect a large number of flow rules in an efficient way. In [114], a framework comprised of actor-based modeling is presented for network verification in SDN. In this framework, the network behaviors are predicted on the basis of the network application’s behaviors and existing model’s correctness properties. The actors in this model are the basic unit of computation, which contain their own memory and have a communication mechanism using asynchronous messages.

In [115], a formal-model-based reverse update mechanism is presented that ensures the maintenance of flow rules characteristics during the transition time in such a way that in-transit packets are processed at the next hops by the same or the latest ACL policy. In addition, it provides a per-packet consistency relaxation concept in the data plane and offers a consistent and efficient policy update technique. This model is compared with two phase update schemes [78], and the results suggest that the proposed model provides much better performance by minimizing overheads while maintaining consistency in flow tables and reducing complexity with the help of wildcard for the composition of flow rules. In communication networks, attackers often attack networks via bandwidth and system/application resource utilization, which leads to the popular denial of service (DoS) attack. How to detect such kinds of attacks is a very interesting research topic in networking. In SDN, deep learning algorithms are implemented according to the information received from the controller to model the attack behaviors. In [116], a distributed DOS (DDOS) attack model is created in Mininet Emulator and Floodlight SDN controller by combining the support vector machine (SVM) classification algorithms. This model detects DDOS attacks with an accuracy rate of 95.24% on a limited number of flow rules. In [117], a network management approach called “Smart-Net System” is proposed in which each data plane device keeps a flow rule in its flow table. If a packet reaches the data plane for the flow rule that exists in the flow table, then it is forwarded to the controller. The controller verifies the behavior of that packet and takes preventive measures to avoid attacks. In [118], a software-defined security (SDS) architecture is presented that is open and universal. It offers an open interface for security services, devices, and management, which is quite helpful for network security vendors to implement network security products and solutions. In this research work, various attack types to which networks can be vulnerable are analyzed, which is helpful for disabling such attacks by modifying the security configuration mechanism at the server.

The trend of increasingly massive IoTs and continuous streaming traffic is driving the demand for increasing computations. Cloud–fog hybrid systems support delay-intensive applications in a distributed computing manner. On the other hand, SDN supports various network infrastructures and inter-controller communication models (flat, horizontal, vertical, hybrid, or T-model) for distributed network management [119]. In addition, the vulnerability and consistency challenge in distributed architecture is more likely to be seen, in contrast to central control. Currently, to support the applications in 5G networks and beyond, the SDN distributed frameworks need to be more sophisticated [120]. Rahman et al. [121] proposed SmartBlock-SDN for efficient resource management and security assurance in blockchain-enabled IoT networks. The proposed framework addresses the challenge of distributed control security and energy-efficient cluster head selection in controllers. SmartBlock-SDN is mapped for a layered approach (IoT, edge, cloud), and cloud-enabled blockchain is considered to cope with the various common network vulnerabilities. Distributed homogeneous controllers and enforced network policies are recorded using immutable blockchains. This stored policy configuration can be accessed using the REST API for various operations in line with network security and resource management. To address flooding attacks, BSDNFilter and blockchain-enabled SDN is proposed in [122]. This work reduces the data packet violations in the SDN network. By employing trust-based filtration, the proposed works outperform a realistic industrial network against network security attacks. In [123], a BMC-SDN architecture is proposed to confine the SDN and blockchain in a network, where control is distributed for failure tolerance and redundant control resources. The proposed work employed blockchain for redundant controllers in various segregated domains. East/west (inter-controller) communication and network operations are recorded using blockchains.

The resource management shortcoming of distributed computational resources (such as NFV, data centers, fog nodes at edges in fog computing) is that energy, storage, and computational resources are limited. With the increase of massive IoT, the 5G and beyond networks will employ more constraints in their resources. 5G mainly has three service use cases: ultra-reliable and low-latency (URLLC), massive machine type computing (mMTC), and enhanced mobile broadband (eMMB). In addition, 6G is a revolutionary initiative in the history of the wireless network, which promises to support a wide geographical region with ultra-high data rates, massive enabled IoTs, connected drones, virtual reality, and network autonomy (by leveraging the machine learning components in the pipeline) [124]. SDN softwarization, southbound interface, east/west interface and on-the-fly management aspects can support the 5G and 6G networks to support a wide area of applications and massive IoTs across the globe. The aspect of resource-limited nodes of edge networks requires efficient resource provisioning in the network for QoS. Phan et al. [125] proposed a dynamic job-offloading mechanism among resource constraints for fog nodes. To enable intelligent offloading for appropriate fog nodes, the SDN controller is utilized, which can support the offloaded task at a minimum cost. The controller can dynamically investigate the resource capabilities, link congestion, and network statistical log files. Using computational offloading among fog nodes decreases the end-to-end latency, traffic detouring to oblivious links, and fog computational resources. In [126], a software-defined network function virtualization (SDNFV) network is presented, in which stateful firewall services are deployed as VNFs to increase network performance, security, and scalability. It utilizes machine learning algorithms to identify potentially malicious linkages and probable attack targets.

To handle a TCP SYN attack, FUPE is proposed in [127], which handles DDOS attacks in a distributed environment. It integrates SDN into its architecture for security objectives. FUPE implements the security-aware task scheduling at the fog gateway. FUPE amalgamates the multi-objective particle swarm optimization algorithm and fuzzy logic for security enhancement. An SDN central resource management unit helps the FUPE with instantaneous decisions in IoT–fog networks. To maintain the security status, FUPE assigns a trusted user’s application tasks to a trustworthy fog computational device in its scheduler architecture. The authors in [128] exploited the SDN and blockchain’s efficiency for network security. Blockchain helps to identify informational alteration at any stage when completed transactions or information are preserved in the form of linked blocks. This study proposes a modified blockchain leveraged with the SDN controller. The SDN controller helps to register the devices in each domain, and the registration information cannot be changed. The SDN controller maintains the public blocks for the registration of devices, while the architecture maintains a private blockchain mechanism at the device level of communication. Each controller is assisted with a blockchain and storage to keep a record of distributed ledgers. Therefore, public and private key-based domain identification of devices supports inter-domain mobility, security, and energy-consumption-aware communication in cyber-physical systems (CPS). The architecture proposed in [129] represents a similar architecture for blockchain-enabled security and energy consumption reduction.

A comprehensive study of SDN-enabled security is conducted in [130]. According to this research, it is hard to tackle cyber-attacks using traditional security mechanisms. The traditional network equipment and network functions cannot support an efficient defense against the attacks because of the network function’s rigidity. The SDN controller supports programmable cyber defense applications in various centralized and decentralized networks. SDN employs detection, localization, proactive, and reactive mitigation against cyber-attacks. SDN controllers can gauge the congestion, port, flow rule entry, and attached end-user device behavior. To ensure security, softwarized control functions of different domains can collaborate and defend against cyber-attacks. DHCPguard [131] exploits DHCP attacks in networks and provides a mechanism to defend against such attacks. It handles the attacks by utilizing the SDN controller—specifically, a security module on top of the POX controller, which is designed for mitigation of DHCP starvation attacks. It also facilitates IP pool recovery, DHCP server availability, snooping, and rate-limiting. The traditional DHCP mechanism lacks a security mechanism (i.e., discovery flooding message of the DHCP client program). Compared to the traditional network forwarding devices, SDN architecture can decide the DHCP client application messages at the central controller and block the suspected or malicious nodes at forwarding devices.

In [132], security assurance is guaranteed through protocol dialects extension. The protocol dialect carries the objectives to provide robustness against downgrade attacks and specializes the network protocol in the context of network security. The OpenFlow protocol dialects have derivatives of MAC-based authentication and complete production packet security without message modifications. In [133], the optimal packet forwarding decisions in fog computing or the optical network need to be reconsidered periodically for efficient network management. Trust and security parameters in fog computing need to be reconsidered in future networks. A malicious fog node can have forged links with other fog nodes and suspicious activities in production packets. It is possible that this sort of node can lock the resources of connected services or alter the topology view at the central SDN controller. Therefore, the resultant computation and energy consumption ratio of fog computing infrastructure also increases. Similarly, in [134], the authors revealed that the current SDN standardization, especially in the form of OpenFlow, needs to be upgraded for flawless integration in fog resources. Fog computing represents a geographical distribution of resources and host applications, which makes the network more vulnerable. To tackle this vulnerability of the fog paradigm, the distributed architecture of the SDN needs to be more defensive.

In [135], vehicular ad hoc networks’ (VANETs) integration with SDN is analyzed. This integration supports efficient resource management for the computational offloading of moving vehicles. Likewise, this is an edge for the various network security issues. VANETs must confront such attacks, such as man-in-the-middle, DDOS, and jamming. If the SDN layer is vulnerable, then the SDN-enabled VANETs are more complex and have poorer defense mechanisms. Moving vehicle applications always trust the nearest roadside unit for computational offloading. In the case of information fabrication or privacy leaks, end-user trust declines for the infrastructure. If the central single control functions are under various attack conditions, then the SDN forwarding devices in VANETs cannot defend and classify the malicious activities or malicious hosts [136]. In IoT-enabled healthcare infrastructures, device authentication is important. The traditional network devices are deficient for authenticating or bootstrapping the fresh connected end devices securely. In [137], the objectives of SDN-supported authentication, routing from the end device to edge server, and inter-edge servers’ communication (routing for the load balance) is analyzed. The proposed framework has an IoT device authentication method that is supported by the probabilistic k-nearest neighbor. The framework uses the probabilistic k-nearest neighbor to evaluate the validity of end IoT devices residing in the healthcare systems. Using p-KNN, an edge server investigates the legitimacy of healthcare IoT devices, and SDN performs efficient collaboration among the edge servers that are close to the computation resources.

To localize the DDOS, a convolution neural network (CNN) is used in [138]. The study claims that the CNN can perform better for DDOS detection compared to logistic regression, multi-layered perceptron, and dense multi-layered perceptron. Furthermore, this work uses game theory to drop malicious activities. As a result, it saves the central SDN controller deployed in any ISP from the IoT devices that are intended for DDOS. If the IoT devices are impairing the central controller, then it increases the vulnerability degree of the SDN-managed ISP. This proposed approach in [139] deals with the SDN security issues to overcome DDOS in a controller and communication switch. It integrates the online learning method to limit the packet-in rate, while tending to the controller queue and switch space capacity. Traditionally, there are straight forwarding approaches to limit the packets rate, but these cannot be trustworthy for bandwidth-sensitive applications in a real network. The proposed parallel online deep learning (PODL) algorithm envisions the two important aspects and adjusts the weight for queuing the controller packets (packet-in) and flow rule installation capacity in a forwarding device.

In [140], SDN-based load-balanced opportunistic routing is proposed for duty-cycled WSNs. In this study, the controller computes and controls the candidates. It prioritizes the candidates by considering the average of three distributions, that is, transmission distance, expected number of hops, and residual energy. It helps to guide the network in such a way that more traffic can flow through the nodes with higher priority. The results show that the proposed approach improves network lifetime, routing efficiency, energy consumption, sender waiting time, and duplicate packets compared with existing approaches. The study in [141] explores the prospects of offloading the 3GPP radio access network (RAN) traffic through WiFi access networks with the help of wireless mesh networks (WMNs). This study reveals an IP wireless mesh network using an SDN-based NFV controller to control and manage the network from the central controller, which results in configuring network devices and services deployment in a fast and effective manner. The study in [142] offers an edge-cloud framework for electric vehicles (EVs). It presents an intelligent network for collaboration between cloud and edge devices to make intelligent decisions regarding charging and discharging of EVs and anticipated demand–supply balance. The proposed solution based on opportunistic SDN (Opp-SDN) exploits the use of EVs in two ways: as energy reservoirs for instantaneous DRM and as forwarding nodes in Opp-SDN.

### Summary and Lessons Learned

The summary of network security and management studies is presented in Table 3. In [110], a token-based authorization mechanism is presented that guarantees exclusive access of network resources to a certain flow for which the user/app has made the reservation. This scheme provides an effective mechanism to ensure exclusive access for network resources. However, resource scheduling and path calculation for flows and the QoS parameter are not considered, which may affect network resource utilization. In [111], network verification is performed, which consists of middleboxes whose forwarding behavior depends on previously observed traffic. It provides a network verification tool that can verify the networks that comprise middle boxes. However, it lacks the ability to verify middlebox code. The research work in [112] helps network administrators so that they can troubleshoot bugs, including root causes of bugs, in their networks to verify that networks are operating correctly. However, it does not suggest integrating the program semantics into network troubleshooting tools or knowledge into network control programs. The research in [115] helps to update and manage network policies in an effective manner. However, it does not investigate the effects of network policy change by analyzing packet violations. The research works in [113,116] detect security issues in the network and warn the network admin to deal with those identified issues. However, the above presented approaches do not deal with the problem when the access rights (network policies) are changed. In addition, these also do not consider the flow rules already installed at the data plane as per old polices. Another SDN approach discussed above is to enable blockchain in the network and record the operations of the network to enhance the network’s security. In particular, in the case of distributed SDN infrastructure(i.e., multiple controllers in cloud-fog infrastructure), blockchain can enhance the network’s efficiency against common vulnerabilities.

The current standardization of the SDN architecture is lacking in terms of security. In particular, ever-increasing delay-aware applications demand vendor lock-in and agnostic security models from various geographical locations in a network. SDN-integrated security can help in centralized and distributed network infrastructure. Although various algorithms are available to make the network secure, mature algorithms are still needed in distributed SDN controller placement. The distributed controller placement makes the SDN-enabled infrastructure more scalable and inclined to the objectives of distributed and fog computing. Security assurance using multiple controller placements in an SDN network requires new solutions for hierarchical, horizontal, and hybrid (T-model) controller synchronization. Similarly, to tackle the various types of attacks in a network, control application autonomy is highly appreciated in ultra-reliable low-latency communication (URLLC). Blockchain technology supports the control layer of the SDN in determining the transactional behavior; however, it can cause end-to-end latency. While machine learning and artificial intelligence can help handle SDN security autonomously and efficiently in 5G and beyond networks, the machine-learning-supported control SDN layer makes the network more reliable. We know that reliability encompasses various performance parameters (i.e., security, failure resiliency, multiple controller placement), but the current era’s main concern is security. With the emergence of fog computing, fog nodes are more vulnerable due to limited resources. On the other hand, it is hard to replicate the control layer applications in fog nodes in a similar context to the cloud. Moreover, local and global (i.e., distributed) network security policies need to be synchronized semantically, which can help to block malicious intentions close to the source network elements. Currently, SDN is lacking in this aspect because the network typologies are in flux in terms of making the SDN-integrated networks more scalable.

## 5. Memory Management Studies

In SDN, flow tables of OpenFlow-enabled switches include controlling functionalities for communication to the SDN controller in addition to flow rule entries for communication in the network. Recent research works revealed that flow rules in data center networks are from 10,000 to 40,000 per second per server rack. In SDN switches, the forwarding table memory is much less than in traditional routers. Most SDN switches have limited on-chip TCAM memory, in which 750 to 2000 flow rules can be stored [144]. These switches utilize a state-of-the-art Broadcom chipset switch that can accommodate 2000 flow rules [145]. This has become big barrier for network management as well as industrialization. The reason behind this fact is that flow tables of these switches are implemented in TCAM due to its better lookup time compared to software-based packet matching. However, TCAMs suffer from large power consumption [146] and expensiveness compared to other memory, for example, static random access memory (SRAM) [147]. The idea is to reduce flow rule entries in the switch flow table by maintaining performance. One approach is to efficiently utilize the forwarding information base (FIB) by compression mechanisms to reduce TCAM requirements. It proposes the ESPRESSO heuristic [148] to minimize the logic to compress prefix-based match fields, which are generated by the optimal routing table constructor (ORTC) algorithm. The simulation results show that FIB size is reduced by 17%, which helps to save TCAMs [149].

Another approach in [150] solves the problem by a flow table reduction scheme (FTRS) by reducing flow table congestion, which helps to reduce flow table size. The simulation results suggest that FTRS reduces flow rules in the flow tables by 98% without compromising network performance and efficiency. In [151], a proactive eviction of flow rule entries is proposed for the efficient utilization of TCAM resources inside OpenFlow-enabled switches. It is based on an intelligent flow management strategy in the SDN controller that combines adaptive idle timeout values for flow rule entries with proactive eviction mechanism on the current TCAM utilization level. In case of non-matching of packets for a defined idle time period, the respective flow rule is removed from the switch flow table. This idle time period is set by the SDN controller before flow rule installation at the data plane. The experimental results show that the proposed scheme, SmartTime, provides 58% better results in terms of cost as compared to static timeout values or random eviction techniques. The authors in [152] investigate the effect of flow rule timeout value based on miss rate performance and flow table occupancy of switches. They observe that with an increase in timeout value, miss rate decreases; however, flow table size increases roughly linearly. They also observe that there is an ideal timeout value, where the miss rate is ideal and the flow table size is also optimal, and with an increase in that particular timeout value, the flow table size increases in addition to its effect on the miss rate. In this research work, a hybrid flow table management mechanism is proposed that combines timeout value with explicit control plane eviction messages. The proposed scheme is able to reduce the flow table size by a lower bound of 57% without affecting the miss rate. However, in the case of TCP-based applications, the flow table size decreases by around 42%.

In addition, this research work analyses the performance of various flow table eviction techniques and finds that the LRU strategy outperforms all others. However, it cannot be implemented in current SDN switches. Moreover, the first-in first-out (FIFO) strategy does not provide better results than LRU, but it is still better than random replacement strategies by 0.1%. The research work in [153] addresses the problem of flow rule placement in firewalls on the basis of ACLs. It aims to reduce the number of flow rules in flow tables of switches by considering conflicts as well as redundancies along with the relationships between neighboring devices. There are two key challenges to implementing it. The first challenge is to check whether a flow rule that is going to be placed in a device is part of a specific rule set or not. The second one is to check whether the flow rule can be merged with the other flow rules or not. This research resolves these challenges by proposing a novel data structure called OPTree to check whether the flow rule belongs to another and whether it can be merged or not. In addition, it proposes flow rule insertion and search algorithms to resolve the identified problem. The results indicate that the proposed approach considerably reduces number of flow rules.

In [154], a mechanism to provide per-flow statistics to the SDN controller is proposed that enhances network performance. The proposed mechanism comprises three phases. In the first phase, a max-flow/min-cost, which is an optimization problem, is formulated to find the optimal forwarding paths. In the second phase, forwarding flow rules for the identified optimal paths are computed via formulating an integer linear problem (ILP) in order to minimize exact-match flow rules in the flow tables of switches to reduce rule-space utilization and to accommodate more flow rules. This is achieved with the help of two greedy heuristic approaches to solve the problem in polynomial time. In the third phase, a flow rule redistribution mechanism is proposed by detecting flow rule congestion at the switches so that new flows can be accommodated in the network. The results of the proposed mechanism are compared with existing mechanisms, such as ReWiFlow [155] and ExactMatch, and show clear improvement in network performance. The research work in [156] proposes a flow rule placement mechanism called “hybrid flow table architecture” that utilizes the advantages of hardware and software flow table implementations. The proposed mechanism handles the decision logic of the placement of flow rules by dynamically placing flow rules in software-based flow tables instead of expensive TCAM modules of switches, without degrading network performance with respect to packet delay or packet loss. Packet classification is very important in networking to perform different tasks (e.g., routing, load-balancing, policy enforcement, etc.).

The research work in [157] proposes a packet classification approach to create packet classifiers based on lossy compression whose representations are semantically equivalent. It helps to find a classifier of optimal size to categorize the network traffic so that appropriately sized TCAM switches can be used for the communication. In [158], fundamental analytical tools are presented that are based on independent sets and alternate paths for better utilizing the TCAMs switches. Moreover, it is useful to validate the optimality of existing coding schemes. In [159], a compression technique is proposed based on random access for forwarding tables. In this mechanism, each forwarding table column is encoded separately via dedicated variable-length binary prefix encoding. The system evaluation reflects that it provides much better results in the compression of forwarding tables compared to existing techniques. In [160], a TCAM update optimization mechanism is presented that ensures consistent packet forwarding. This mechanism is based on a modified-entry-first write-back scheme that considerably decreases TCAM entries’ movement overhead and detects reordering cases with the help of efficient solutions.

### Summary and Lessons Learned

The summary of memory management studies is presented in Table 4, which describes the efficient utilization of TCAM resources of switches. In [148], an ESPRESSO heuristic approach is proposed, which is based on the optimal routing table constructor (ORTC) algorithm to optimize routing table size for better utilization of TCAMs. This mechanism is quite effective in traditional networking; however, it needs to be tested in an SDN environment. The research in [151] presents a flow management strategy in an SDN controller that offers a proactive eviction mechanism in TCAMs by preventing table misses at an optimum level. This work is based on idle timeout values for flow rules and does not consider other parameters, such as initial idle timeout, max idle timeout, or rate of timeout increase, which are quite helpful in flow rule management. In [152], a hybrid flow table management scheme is presented that combines timeout value with explicit control plane eviction messages by considering both the miss rate and flow table size. This strategy is implemented by using same timeout values for all flow rules, and dynamic timeout values are not considered, although they impact real-time networks.

OPTree [153] and FlowStat [154] represent two flow rule placement strategies to reduce the number of flow rules in the flow tables of switches by considering the relationships between network devices. In addition, the flow rules are installed on optimal paths to avoid network congestion. These approaches lack an investigation into the network topology and policy change to install flow rules. In [157,159], flow rule compression mechanisms are proposed for efficient memory management of switches by classifying the network traffic to accommodate more flow rules. However, these approaches do not consider flow rules compression based on a limited-size longest prefix match classifier, which can correctly classify a high portion of traffic. In addition, managing massive flows in limited-size switch flow tables remains a challenge. Moreover, proactive flow rule installation in delay-sensitive applications and non-delay applications or best-effort traffic should benefit from reactive flow rule installation. It is yet another interesting research area to explore and propose a scheme considering traffic variability.

## 6. SDN Simulators and Emulators

To analyze the network performance, instead of implementing a large experimental testbed, there are two commonly used methods; these are called simulation and emulation. The simulation method provides an application environment where we can test our implemented software program without real deployment. The emulation method utilizes a software program to perform executions with real devices by interacting with them as when required. To analyze network performance by simulation is inexpensive, flexible, controllable, and scalable compared to an emulator. In addition, the simulators allow researchers to analyze and test network behaviors as per defined workload. In SDN, with the development of OpenFlow protocol, the simulation tools have extended support to additional network components for the testing and experimentation of OpenFlow-based network applications. Moreover, network emulators based on software switches have also been developed to test and analyze network applications, for example, Open vSwitch (OvS) [161], ofsoftswitch13 [162], Indigo Virtual Switch (IVS) [163], and so on.

The Mininet SDN emulator [164] offers a rapid prototyping workflow and virtualization functionalities along with command line interface (CLI) and API on one physical machine that allows network developers to configure, manage, and test their networks. It helps to create a network topology for a network scenario that consists of virtual hosts, switches, links, and controller platforms. It supports research and development, learning, prototyping, testing, debugging, and any other task related to network experimentation on a computer. In the basic implementation of Mininet, the performance fidelity is not included. In Mininet-HiFi [165], these improvements are implemented. It also has a cluster edition prototype [166], and other releases include Maxinet [167] and Mininet-CE [168], which fixes the limitations of large-scale implementation of SDN emulations. Finally, two experimental frameworks for SDN data centers are also developed; these are datacenter in a box [169] and SDDC [170]. The distributed OF testbed (DOT) [171] is a highly scalable emulator that provides an emulated network across a cluster of computers that guarantees computation and network resources to switches, hosts, and links.

The OFNET [172] emulator provides built-in functionalities to test and debug, and traffic generation and monitoring tools, which help researchers in the debugging process. The virtual network overlay (ViNO) [173] network emulation tool provides functionalities that help to create arbitrary network typologies via Open vSwitches and virtual machines. The overlay interconnection between virtual machines (VMs) is provided by VXLAN encapsulation [174]. EstiNet [175] provides the benefits of both simulation and emulation tools by offering each host a real Linux OS environment, and any real application program can run on a simulated host without any modification. FS-SDN [176] is a simulator that is based on the FS [177] simulation platform and is built in Python language. OMNeT++ [178,179] is a network simulator that is developed in the C++ language for network modeling, multiprocessors, and different distributed or parallel systems. It utilizes the INET framework [180] for the simulations in the SDN environment by integrating OpenFlow components, basic switch functions, basic controllers, and OpenFlow messages. The NS-3 network simulator [181] is implemented in C++ and can use OpenFlow switches. In addition, it also offers the use of external modules to extend NS-3, such as OFSwitch13 [182], which helps for OpenFlow 1.3 compatibility.

### Summary and Lessons Learned

The summary of network simulators and emulators is presented in Table 5. It provides a comprehensive overview of simulators and emulators that are developed in various programming languages, such as C, C++, Java, Python, and so on. These tools help in developing and simulating SDN applications in an SDN environment. Mininet [164] is an innovative emulation tool that allows network developers to configure, manage, and test their networks. These networks cannot exceed single-server bandwidth and cannot run non-Linux-compatible OpenFlow switches or applications. The DOT [171] emulation tool ensures computation and network resources for switches, hosts, and links in large SDN deployments. It can be used in an environment where a fixed number of physical machines are used to emulate a given network and does not support dynamic scalability and multi-user support. OFNET [172] is an open-source emulator to test and debug networks for analyzing network traffic. This tool does not support cloud platforms and large-scale implementation of layer-2 networks. ViNO [173] helps to create arbitrary network topologies via Open vSwitches and virtual machines. However, its scalability and OpenFlow support are not specified, and these can be helpful for experimentation and testing.

The EstiNet [175] network emulator provides accuracy, quickness, repetition, and scalability and is based on a kernel-reentering simulation methodology that allows researchers to test their applications. The FS-SDN [176] simulator is developed in the Python language for realistic testing and validation of standard networks. It can be extended for debugging and tracing capabilities, which can be helpful for developing new SDN applications. The OMNeT++ [178,179] simulator is helpful for network modeling, multiprocessors, and different distributed or parallel systems. However, its kernel is implemented in C++ and can only run with a modern C++ compiler. The NS-3 network simulator provides the capability to add new protocols and allows integration and customizability without remaking the core of the simulator. However, it is lacking a visual interface for creating a topology and for visible capability on an experimental level. This study provides a feature-based comparison of SDN simulators and emulators including a brief description, implementation language, and the strengths and weaknesses. It can be extended to conduct a performance-based comparison of these tools under specific scenarios. Finally, new simulators and emulators need to be developed for hybrid SDN, wireless networks, fog/edge computing, cloud computing, and so on, to meet the needs of future networks.

## 7. SDN Programming Languages

SDN programming languages consist of compilation and validation tools that are helpful for the translation of high-level constructs into messages understandable by the SDN controller API. The following section explains some SDN programming languages. One of the languages is Frenetic [183,184], which is a high-level language for the programming of OpenFlow networks and is useful for the categorization and accretion of network traffic. Moreover, it is also helpful for defining packet forwarding policies on the basis of a functional reactive combinator library inspired by Yampa [185], and its implementation is based on FlapJax [186]. By providing a Frenetic runtime environment, facilities pertaining to installation and querying low-level details are managed. In addition, it provides compositional constructs that facilitate modular reasoning and enable code reuse. As NetCore [187] is the successor of Frenetic, it carries an enhanced policy management library. Moreover, it has the capacity to compile ACL policies and handle the interaction between controller and switch. In addition to this, for the efficient generation of flow rules, the run-time system of NetCore is designed. Nettle [188] is a low-level programming language that deals with streams and does not deal with events. It is quite appropriate for various functions such as programming controllers, and programming discrete and continuous operations. Moreover, dynamic policies, including traffic engineering and load balancing, are also generated through it. As it is declarative in nature, functions that are time sensitive and varying can be demarcated. Moreover, the sequential operator provided by Nettle can also be used for creating compound commands.

Procera [189] is a high-level programming language that is helpful to delineate ACL policies in communication networks. It is quite a resource for the operators as it provides an expressive and extensible compositional framework. Moreover, it is also quite useful for designing network applications that not only react to the events produced by OpenFlow switches but also to external events, for instance, user authorization and bandwidth usage. Procera was used in several campus networks, as well as home network prototype deployments [190]. Flow-based management language (FML) [191] is a high-level declarative programming language that is based on non-recursive Datalog [192] for handling a network whose aim is to provide efficient and flexible policies. Moreover, it is also helpful for the operators, as it provides them with eminent management facilities for configuring ACL policies straightforwardly. Flog [193] is an event-driven programming language that adopted ideas from FML and Frenetic and is based on logic programming for the SDN environment, similar to FML. It is composed of three components that are similar to Frenetic. These components include a mechanism for network state collection, information processing, and policy generation. Like NetCore, Frenetic-OCaml [194] is also a successor of Frenetic. It is beneficial in providing mechanisms for network-wide policy implementation. NetCore is used and is replaced with NetKat for forwarding decisions. Moreover, its query language permits querying statistics that include traffic and topology. Being an imperative programming paradigm-based language, Pyretic [195], by specifying the static and dynamic forwarding policies, assists in developing network applications. By utilizing the sequential and parallel operators provided by Pyretic, the forwarding policies can be specified.

FlowLog [196,197] is a declarative language for programming SDN network applications. Being a finite state language, for the various types of analysis, the model checking can be applied quite competently. FlowLog has two versions. One is based on NetCore, whereas the other is built on the packet-handling capability of Frentic-OCaml. The FatTire [198] SDN programming language is used for writing fault-tolerant network applications. It is designed for the purpose of specifying the path for packet routing and fault tolerance. Moreover, it can also be helpful for the programmer who, by using regular expressions, declaratively states the sets of necessary paths. NetKat [199,200,201,202] uses Kleene algebra with tests (KAT) [203]. This programming language is based on equational theory, for programming and reasoning about the networks. A regular expression can be used for describing end-to-end paths, and its semantics are inspired by NetKat. Moreover, NetKat is also beneficial for defining virtual topologies. Merlin [196,197,204,205] is a declarative language that is useful for distributing and managing the ACL policy implementation process. Its run-time monitor is used to examine incoming and outgoing network traffic. Being a policy specification for SDN, PonderFlow [198] aims to extend the Ponder language [206] for describing OpenFlow flow rules. It is used to define management and security policies in distributed systems.

PonderFlow provides mechanisms for implementing access control and network abstractions. NOF [207] is a programming language with the objective of enabling network application to design the network according to the application requirements. It comprises sets of operations and services, and these are categorized into three groups, namely matching, timing, and query. Operations can be applied to conventional network fields that are based on host information. Timing includes information about the services, that is, when they will be installed and how long they will remain functional in the network. Query operation helps to obtain the network state information, that is, link state, bandwidth usage, transmission errors, and so on. Kinetic [208] is a domain-specific language that helps network operators control the dynamic state of their network. In addition, it offers facilities to validate the accuracy of control programs. The network policies may be stated with respect to finite state machines (FSMs), which aids in encapsulating the dynamic state of the network.

### Summary and Lessons Learned

The summary of SDN programming languages is presented in Table 6. Different SDN programming languages are developed to handle specific problems or to provide specific functionalities in network applications in a more refined and abstract manner. Most of the languages, (i.e., Procera [189], Pyretic [195], FlowLog [209,210], FatTire [157], NetKat [158,159,160,161]) provide basic-level flow matching. Pyretic [195] implements native flow matching and virtualizations, unlike other languages. Frenetic-OCaml [194] and Frenetic [183,184] provide enhanced monitoring based on query language and windowed history as well as flow matching. Kinetic [208] provides inherited flow matching and monitoring services, and it implements the modules in a parallel fashion. We have described these languages along with their programming paradigms.

These languages help network administrators to implement access control, and to develop and test network applications on the basis of low-level constructs as well as high-level abstractions. However, there is still a wide scope for researchers to offer new abstractions and also contribute to the advance of NBI standardization. The future programming languages may include functionalities of load balancing to avoid congestion on specific resources. Researchers may be interested in using language constructs to scale-up the resources in an elastic way. Moreover, future programming languages should incorporate NFV to manage the virtualized functions and OpenFlow updates to take advantage of the new features offered by OFP. Network forensics are quite useful to collect information on network devices to verify the evidence of crimes. However, this important area is also lacking in research with regard to SDN programming languages. Finally, the current programming languages still do not provide an open interface to allow new modules to be developed and incorporated into the language, which is also an interesting future research area.

## 8. SDN Controller Platforms

The controllers are the brains of SDN networks and act as a strategic control point. These contain collection of modules that can perform different network tasks including network topology, network statistics, and so on. Different network applications such as network policies are installed on the controllers for data communication between end nodes. In this section, we discuss some controller platforms, which are described below.

Beacon [212] is implemented in the Java programming language and uses centralized architecture. Moreover, it uses ad hoc northbound API and southbound API with OpenFlow 1.0, which supports CLI and web user interface (WebUI). It also supports multi-command-line threading and modularity functionality. It serves as the basis of Floodlight, with a focus on being developer friendly and high performance, and with the ability to start and stop existing approaches. It has explored areas of OpenFlow controller design. Beehive [213] is a distributed control platform implemented in the GO programming language with a distributed hierarchical architecture. It utilizes REST northbound API and southbound API with OpenFlow 1.0 and 1.2. It utilizes the Linux supporting platform and supports CLI. The implementation of DCFabric [214] is based on the C and Java script programming languages, and it has a centralized architecture. It utilizes the REST northbound API and southbound API with OpenFlow 1.3. It uses the Linux supporting platform and supports CLI and WebUI. Moreover, it supports multi-threading and has a good modularity functionality with good consistency. Disco [215] is implemented in the Java programming language with a distributed flat architecture. It utilizes the northbound, southbound, and east/westbound API with REST with OpenFlow 1.0 and AMQP, respectively. It supports proprietary licenses. It has good modularity with limited documentation. The implementation of Faucet [216] is based on the Python programming language, and it has a centralized architecture. It utilizes SBI with OpenFlow 1.3. It employs the Linux supporting platform and supports CLI and WebUI. It supports Apache 2.0 licenses and multi-threading with good consistency.

Floodlight [217] is implemented in the Java programming language, and it has a centralized architecture. It utilizes REST, Java, RPC, and Quantum northbound API and southbound API with OpenFlow 1.0 and 1.3. It utilizes a Linux-, MacOS-, and Windows-supporting platform and provides CLI and WebUI. It supports Apache 2.0 licenses, which supports multi-threading, and has a fair modularity with good consistency and documentation. Flow Visor [218] is implemented in the C programming language with a centralized architecture. It utilizes JSON and RPC northbound API and southbound API with OpenFlow 1.0 and 1.3. It utilizes a Linux supporting platform and supports CLI interface. Moreover, it supports proprietary licenses and has no consistency; however, its documentation is fair. HyperFlow [219] is implemented in the C++ programming language with a distributed flat architecture. It utilizes SBI with OpenFlow 1.0 and east/westbound API with publishing and subscribing messages. It supports proprietary licenses and multi-threading and has no consistency. Kandoo [220] is implemented in the C, C++, and Python programming languages, and it has a distributed hierarchical architecture. It utilizes Java RPC northbound API and SBI with OpenFlow 1.0–1.2 and east/westbound API with messaging channel. It utilizes a Linux supporting platform and supports CLI and proprietary licenses.

Loom [221] is implemented in the Erlang programming language and has a distributed flat architecture. It utilizes JSON NBI and SBI with OpenFlow 1.3–1.4. It utilizes a Linux supporting platform and supports CLI. It supports Apache 2.0 licenses and multi-threading and has a good modularity with good consistency. However, its documentation is limited. Maestro [222] is implemented in the Java programming language with a centralized architecture. It applies ad hoc northbound API and southbound API with OpenFlow 1.0. It utilizes Linux, MacOS, and Windows supporting platform and supports WebUI. It supports LGPL 2.1 licenses. It supports multi-threading and has a fair modularity with no consistency, and its documentation is also limited. MsNettle [223] is implemented in the Haskell programming language and has a centralized architecture. It utilizes southbound API with OpenFlow 1.0. It utilizes a Linux supporting platform and supports CLI. It supports proprietary licenses and multi-threading and has a good modularity with no consistency. Meridian [224] is implemented in the Java programming language and has a centralized architecture. It utilizes REST NBI and SBI with OpenFlow 1.0 and 1.3. It utilizes a cloud-based supporting platform and supports WebUI. It supports multi-threading and has a good modularity with no consistency.

Microflow [225] is implemented in the C programming language with a centralized architecture. It utilizes Socket NBI and SBI with OpenFlow 1.0–1.5. It utilizes a Linux supporting platform and supports CLI and WebUI. It supports Apache 2.0 licenses and multi-threading. Nodeflow [226] is implemented in the JavaScript programming language with a centralized architecture. It utilizes JSON northbound API and southbound API with OpenFlow 1.0. It utilizes a Node.js supporting platform and supports CLI. NOX [227] is implemented in the C++ programming language and has a centralized architecture. It utilizes ad hoc NBI and SBI with OpenFlow 1.0. It utilizes a Linux supporting platform and supports CLI and WebUI. It supports GPL 3.0 licenses and multi-threading (Nox-MT) and has low modularity with no consistency. ONIX [228] is implemented in the C++ programming language with a distributed flat architecture. It utilizes Onix API northbound API and southbound API with OpenFlow 1.0 and OVSDB and east/westbound API with Zookeeper. It supports proprietary licenses and multi-threading and has a good modularity with no consistency.

ONOS [229] is implemented in the Java programming language with a distributed flat architecture. It utilizes REST and Neutron NBI and SBI with OpenFlow 1.0 and 1.3 and east/westbound API with Raft. It utilizes a Linux, MacOS, and Windows supporting platform and supports CLI and WebUI. It supports Apache 2.0 licenses and multi-threading functionality and has a high modularity and consistency. OpenContrail [230] is implemented in the C, C++, and Python programming languages with a centralized architecture. It utilizes REST NBI and SBI with BGP and XMPP. It utilizes a Linux supporting platform and supports CLI and WebUI. It supports Apache 2.0 licenses and multi-threading functionality and has a high modularity with good consistency. OpenDaylight [231] is implemented in the Java programming language with a distributed flat architecture. It utilizes REST, RESTCONF, XMPP, and NETCONE NBI and SBI with OpenFlow 1.0 and 1.3 and east/westbound API. It a utilizes Linux, MacOS, and Windows supporting platform and supports CLI and WebUI. It supports EPL 1.0 licenses and multi-threading functionality and has a high modularity with consistency. OpenIRIS [232] is implemented in the Java programming language with a distributed flat architecture. It utilizes REST NBI, SBI with OpenFlow 1.0–1.3, and east/westbound API with custom protocol. It utilizes a Linux supporting platform and supports CLI and WebUI. It supports Apache 2.0 and multi-threading functionality, with fair modularity and no consistency. OpenMul [233] is implemented in the C programming language and has a centralized architecture. It utilizes REST NBI and SBI with OpenFlow 1.0, 1.3, OVSDB, and Netconf. It utilizes a Linux platform and supports CLI. It supports GPL 2.0 licenses and multi-threading functionality and has a high modularity with no consistency. However, its documentation is good.

PANE [234] is implemented in the Haskell programming language with a distributed flat architecture. It utilizes PANE NBI, SBI with OpenFlow 1.0, and Zookeeper east/ westbound API. It utilizes Linux and MacOS platforms and supports CLI. It supports BSD 3.0 licenses and has a fair modularity with no consistency, but with documentation. POF Controller [235] is implemented in the Java programming language and has a centralized architecture. It utilizes SBI with OpenFlow 1.0 and POF-FIS. It utilizes a Linux platform and supports CLI and WebUI. It supports Apache 2.0 licenses, and its documentation is limited. POX [236] is implemented in the Python programming language with a centralized architecture. It utilizes ad hoc NBI and SBI with OpenFlow 1.0. It utilizes a Linux, MacOS, and Windows platform and supports CLI and WebUI. It supports Apache 2.0 licenses; however, it does not support multi-threading functionality and has a low modularity with no consistency. Ravel [237] is implemented in the Python programming language and has a centralized architecture. It utilizes ad hoc NBI and SBI with OpenFlow 1.0. It utilizes a Linux platform in CLI mode. It supports Apache 2.0 licenses, and its documentation is fair.

Rosemary [238] is implemented in the C programming language with a centralized architecture. It utilizes ad hoc NBI and SBI with OpenFlow 1.0, 1.3, and XMPP. It utilizes a Linux supporting platform and supports CLI. It supports proprietary and multi-threading functionality and has a good modularity with no consistency. RunOS [239] is implemented in the C++ programming language with a distributed flat architecture. It utilizes REST NBI, SBI with OpenFlow 1.3, and Maple east/westbound API. It utilizes a Linux supporting platform and supports CLI and WebUI. It supports Apache 2.0 licenses and multi-threading functionality and has a high modularity with consistency. Ryu [240] is implemented in the Python programming language and has a centralized architecture. It utilizes REST NBI and SBI with OpenFlow 1.0–1.5. It utilizes a Linux and MacOS supporting platform in CLI mode. It supports Apache 2.0 licenses and multi-threading functionality. SMaRtLight [241] is implemented in the Java programming language with a distributed flat architecture. It utilizes REST NBI, SBI with OpenFlow 1.3, and BFT-SMaRt east/westbound. It utilizes a Linux supporting platform in CLI mode. It supports proprietary licenses and has no consistency. TinySDN [242] is implemented in the C programming language with a centralized architecture. It utilizes SBI with OpenFlow 1.0 and a Linux supporting platform in CLI mode. It supports BSD 3.0 licenses and has no multi-threading functionality or consistency. Trema [243] is implemented in the C and Ruby programming languages with a centralized architecture. It utilizes ad hoc NBI and SBI with OpenFlow 1.0. It utilizes a Linux supporting platform in CLI mode. It supports GPL 2.0 licenses and has good modularity, but it has no consistency. However, its documentation is fair. Yanc [244] is implemented in the C and C++ programming languages and has a distributed flat architecture. It utilizes REST NBI and SBI with OpenFlow 1.0–1.3 capabilities. It utilizes a Linux supporting platform and supports CLI. It supports proprietary licenses, and its documentation is limited. ZeroSDN [245] is implemented in the C++ programming language and has a distributed flat architecture. It utilizes REST NBI, SBI with OpenFlow 1.0 and 1.3, and ZeroMQ of east/westbound API. It utilizes a Linux supporting platform in both CLI and WebUI modes. It supports Apache 2.0 licenses and has high modularity with fair documentation.

### Summary and Lessons Learned

The summary of SDN controller platforms is presented in Table 7. These controllers manage flows to the switches/routers via SBI and the applications/business logic via NBI to deploy intelligent networks. The controllers install flow rules at the data plane devices (switches/routers) to perform required functionalities, such as forwarding, dropping, and so on. There are different kinds of SDN controllers (centralized and distributed), which are developed to perform various functionalities in different programming languages (Python, C, C++, Java, etc.). Some of the controllers are java-based (i.e., Beacon [212], Disco, Opendaylight, SMaRtLight [241], etc.). Some of them are based on distributed approaches. Ryu is very easy and straightforward to program. Beginners can easily deploy and use this controller for their network.

Opendaylight is complex, and it is difficult to model new ideas. For experts, it is good option to use because it provides a dozen southbound APIs and protocols, such as NetConf, OVSDB, and PCEP, for managing and configuring forwarding devices. If someone want to rank these controllers on the basis of simplicity, the order would be as follows: Ryu, Floodlight, ONOS, ODL. The scalability, consistency, reliability, and security are very important to consider in designing an efficient and robust SDN controller. The current SDN controllers lack standard data models, anomaly detection, and security mechanisms. It is observed that developing a brand new SDN controller may not be the best solution; however, the existing SDN control frameworks need to be enhanced, refined, and improved to address the above-mentioned issues.

## 9. Comparison with Existing Studies

SDN is a new networking paradigm that influences the network operations and management, gaining the attention of the research community and other organizations [246]. The papers have been written to discuss different issues and challenges of SDN, and these are shown in Table 8. Akyildiz et al. [247] wrote a survey related to fault management, fault tolerance, topology update, and traffic analysis. They explained different aspects of the SDN architecture and their interactions. Several studies are classified according to their specific problems. Xie et al. [248] compile techniques such as routing optimization, QoS, and resource management security to fill the gaps left by the previous paper. Their main focus was on blending the QoS-aware techniques and presenting their work in a comprehensive manner. Meanwhile, Matlou et al. [249] surveyed the same topic to improve on the paper written by Xie et al. [248]. They targeted wireless sensor networks and SDNs to explain this topic. They achieved their target, as wireless networking in SDN was considered a unique topic at that time, and they accepted the challenge to complete the survey. In the same year, Jose et al. [250] wrote a comprehensive review related to traffic classification and security. Network intrusion detection is also considered as a point in traffic engineering, and it was not included in any of the papers mentioned above, so to describe it in a comprehensive manner, Sultana et al. [251] focused on the four learning algorithms included in the paper. Similarly, traffic profiling is also an important area of traffic classification. Cui et al. [252] considered all four learning algorithms and explained several machine learning models, including traffic profiling and its functioning in SDN networking. It was considered an important topic at that time due to its uniqueness. Moreover, Loung et al. [253] focused on the topic of network virtualization, which was explained and included in a survey related to traffic engineering. They achieved their target by reviewing network virtualization and ensuring QoS in virtual networks.

Kreutz et al. [254] started their discussion by defining the SDN, its major concepts, and its differences compared to traditional networks. The architecture of an SDN is presented in a bottom-up approach. Deep analysis is performed at its architecture, APIs, network programming, and network layers. They also focused on the major problem of cross-layering and its solutions. Keeping in view security, performance, scalability, and resilience, the design of the controller and switches are addressed in this study. Wang et al. [255] provide a review of diverse problems in networking, including traffic classification, traffic prediction, self-configuration, and network management, as well as performance inspection and prediction. They focused on a small number of studies to showcase different aspects of the workflow. Jamshidi et al. [256] explained applications based on machine learning (ML) methods and techniques by dividing them into six categories of networking, which are traffic prediction, network security, cloud services, application identification, domain name system, and QoS. For all these categories, they determined the ML methods and input datasets. They also summarize the various challenges and major findings of these input data and ML methods. In particular, they discovered multiple new aspects of ML in networking. They ended their study by discussing research gaps and challenges. Mohammed et al. [257] review existing different ML and deep learning (DL) algorithms in the context of SDN networking for the measurement of traffic classification and traffic prediction. DL approaches are used for traffic prediction. Tam et al. [258] directed their attention to ML-based security solutions for SDN. ML models used in network prediction and prevention are identified to be deficient, so attackers can control or avoid the model. Attackers are also versed in ML capabilities to predict the defending model’s behavior. The authors suggest some specific recommendations that are helpful for SDN security. They recommend that a secure development process must be followed. They made an auditable ML model. This is important to give attention to threat models, instead of scheming ML solutions, and there must be an operational cost model that is produced at the initial level. These recommendations are helpful to improve the properties of ML-based solutions for SDN.

Amin et al. [259] discuss the deployment of SDN among legacy networks. Due to the speedy growth of the Internet, network structures have become huge and complicated. This complexity initiates a huge amount of traffic data, and it becomes a challenge to take traffic measurements such as traffic classification and prediction, in a network. To manage networks efficiently, the SDN paradigm is adopted, and it has already been adopted by several organizations. This survey presents a comprehensive study on a specific topic. For hybrid SDN, some efficient algorithms are needed to measure and deploy the SDN alongside traditional networking. Priyadarsini et al. [260] provide a comprehensive review and report of state-of-the-art progress on productive traffic management, including load balancing and energy-efficient routing. The introduction and deployment of SDN controls, network safety, and optimum positioning of controllers affecting traffic management are also discussed. This paper also addresses a few unexplored SDN challenges, such as modular implementation, convergence with the legacy network, and possible analysis charts. Although there are multiple studies on SDN, most of them are old and do not cover the state-of-the-art approaches. Some of them just cover one or two aspects of the SDN environment (i.e., SDN controller placement [261,262], SDN programming languages [263,264], SDN simulators [265,266]).

In all of the above-mentioned studies, none of them evaluate the state-of-the-art approaches related to different classifications of SDN. We categorize the approaches as network testing and verification, flow rules installations, SDN controllers, SDN simulators, network security and management, programming languages, and memory management. Moreover, we provide comparisons of all these categories in the form of tables and discuss limitations of each technique, which require attention in future research. In SDN, network devices are controlled using flow rule installations, and there are several methods for rule installations in different circumstance (i.e., path failure, new rules, rules update). This is an important aspect that needs to be considered for evaluation of different approaches. Network devices in SDN are equipped with TCAM memory, which is very limited. It is necessary to use this memory very efficiently, so many approaches are adopted for memory management in SDN devices. We cover all these studies and compare their performance and efficiency.

## 10. Future Research Directions

This paper focuses on different techniques found in OpenFlow-based SDN. We observe that most existing techniques that appear in the literature complement the methods and subordinate them. Following are the possible future research directions for the research community. SDN controllers handle communication traffic, and as network traffic grows, the mapping of flow rules between controller and switches becomes overburdened, while a few controllers become unburdened. Due to poor throughput and long reaction times, such an imbalance impairs the performance of the SDN network. It is difficult to manage the load across several controllers dynamically. In SDN security, several SDN-based defensive primitives are addressed. To cope with contemporary cyber dangers, both novel primitives and modular protection systems that employ various primitives are required. Researchers should focus on improving the security of established network protocols (such as address resolution protocol (ARP), dynamic host configuration protocol (DHCP), domain name system (DNS), etc.), as well as novel protocols such as neighbour discovery protocol (NDP) in Internet Protocol Version 6 (IPV6).

With respect to controller scalability, separation of control and data planes, quantity of events/requests handled by a controller, and controller–switch communication delay are all bottlenecks in SDN. The relationship between different approaches used to optimize controllers and scalability difficulties are also examined. The majority of current storage systems have been evaluated on small-size networks, such as those with 7–28 nodes and 7–43 connections. As a result, these figures are incomparable to a large number of devices in a wide-scale network environment, such as Telco, Internet of things (IoT), and so on. In resource management (i.e., network components such as switches and controllers), ML approaches are extremely successful. The majority of ML algorithms, on the other hand, are focused on flow categorization and monitoring. Much less study has been done on estimating traffic flow for real-time applications and best-effort traffic and deciding which traffic flow to install ahead of time. These are multiple research areas to explore. In addition, the energy-efficient SDN networks, wireless networks, network virtualization techniques, cloud computing platforms, and SDN migration mechanisms are recommended for more detailed exploration.

Information-centric networking (ICN) [267] is gaining popularity for the future Internet to increase the efficiency of content delivery and availability. With the increasing demand for video streaming of public users through mobile devices, the Internet speed is significant to fulfill the desired need for end-users through efficient use of bandwidth. Nowadays, different applications of ICN are very popular, such as SDN-based ICN [268], IoT-based ICN [269], ICN with edge computing [270], green ICN [271], and so on. These areas need to be explored in more detail to help the ICN community reach the next step of implementation in live deployments. The network programmability feature of SDN is used to enrich response functionality. The data plane provides the possibility of adding new functions that are more competent to secure the entire network. In prevention systems, security policies are defined to stop attackers from contacting targets, which require investigation once policies change. The dynamic flow control features of SDN enhance the detection of attacks without adding middleboxes and virtually turn switches into network security devices that can prevent attacks dynamically. Moreover, ML-based SDN includes network optimization, improving network security, and high-quality training datasets. Some other broader perspectives on SDN, such as software-defined mobile networks and software-defined vehicular networks, are also important areas to explore. Regarding QoS, researchers are carrying out experiments with real matrices through different network topologies so that each flow may have different QoS requirements. If DROM [272] is extended with QoS routing, more efficient and enhanced results can be generated. The QoS measures the traffic conditions and traffic classification, while DROM dynamically measures the reliability, effectiveness, and awareness of QoS. The queuing delay of the switches and the processing delay of the server improves the QoS.

For traffic engineering, some machine learning techniques provide fundamental improvements compared to the traditional traffic engineering paradigm. Many researchers have devoted their skills to developing efficient systems for traffic classification, routing, and traffic optimizations. Network policies are formed intelligently using some machine learning algorithms, for example, random forest, support vector machine (SVM) [273], k-nearest neighbors (KNN), and so on. Apart from machine learning, deep learning models such as the artificial neural network (ANN) or convolutional neural network (CNN) may also be adopted. For example, if we can predict the change in network policies before its occurrence and take the needed measures, then much better throughput can be achieved. These approaches will make the controller more intelligent, which results in more efficient handling of network policy change phenomena. Moreover, quality of service (QoS) parameters also need to be considered to manage the network traffic to improve network throughput. However, machine learning algorithms have their own limitations, such as false negatives [274]. For traffic classification, most of the research conducted so far has been on labeled datasets using a supervised learning approach. Few works are done using semi-supervised learning, where some of the data are labeled and some are not given labels. The same applies to the scenario with unsupervised learning. Another important learning approach is reinforcement learning, which is a black box approach when we consider traffic classification. In this regard, algorithms can be designed to classify the data traffic in such a way that the new classified information can help the algorithm learn from the experience.

## 11. Conclusions

The SDN architecture has shifted network control and management to a centralized controller, which provides a variety of benefits including programmability, innovation, and ease of security policy implementation. In this paper, we briefly reviewed the traditional networking and SDN structure along with its background, application programming interfaces, network configurations, and benefits of the SDN paradigm. Afterwards, we organized this paper into seven groups, namely network testing and verification, flow rule installation mechanisms, network security and management issues related to SDN implementation, memory management studies, SDN simulators and emulators, SDN programming languages, and SDN controller platforms. We discussed each category in detail along with the implementation mechanisms and analyzed these mechanisms by summarizing and comparing each technique along with the lessons learned from the proposed techniques. Furthermore, we analyze and discuss the latest studies and compare these papers with our research paper. Finally, comprehensive future research guidelines are provided, and the paper is concluded.

## Figures and Tables

**Table 1:** Summary of network testing and verification studies.

Studies	Techniques	Description	Strengths	Weaknesses
NDB [70]	Mininet	Tracks down root causes of bugs	Breakpoints and packet backtracking	Debugging time overhead
VeriFlow [72]	NOX, Mininet	Checks network invariants in real time and prevents faulty rules	Flow rule debugging for reachability analysis	Not suitable for delay-sensitive and QoS-constrained applications
Flow Checker [75]	OpenFlow switch, flow table	Verifies flow tables based on behaviors of flow rules	Inconsistencies localization in device flow tables	Can only be used in small network
Anteater [76]	Linux, C++, Ruby	Examines the state of data plane and verifies network invariants	Control plane configuration analysis	Inconsistent data plane map generation for dynamically changing FIBs
NICE [81]	Mininet, OpenFlow switch, Network X	Utilizes model checking and symbolic execution for bug investigation	Simplification of switch modeling and event testing	Unable to test a controller implemented in the same language
FPB [82]	Python, OpenFlow, NOX	Provides a formal model for consistent policy update	Least controller intervention	Buffering ability in case of switch to controller link failure is not discussed
HSA [83]	Ubuntu, flow-based management language, Prolog	Protocol-independent static network-invariant investigations	No need to modify the protocol for implementation of HSA	Static space analysis mechanism
Py-Resonance [84]	Pyretic, Python	Utilizes state-based policies to predict network’s behaviour	Modular network function control	TCAM under- utilization by least significant policy states in FSM model
PGA [85]	Mininet, Pyretic compiler, POX controller	Composition of ACL policies that inspects multiple policies	Conflict-free forwarding rule translation	Scalability issues and support of HW/VM middleboxes
SFC [87]	Java-based prototype, OpenFlow switch	Identifies the anomalies in ACL policies before deployment	Proactive anomalies detection independent of programming language	Overhead for generating flow rules at data plane

**Table 2:** Summary of flow rules installation mechanisms.

Studies	Techniques	Description	Strengths	Weaknesses
ORPP [88,89]	Mininet, OpenFlow	Resolves the offline and online ORPP problem for the known set of flows, which varies over time	Flow rule prioritization and optimal placement	Does not consider the forthcoming load in low priority path/flow rules
vCRIB [90]	VM, Open vSwitch, TCAM	Provision of an abstraction for specifying and managing flow rules by automatic partitioning	Considers cost-effective resource utilization and machine performance constraints	Low scalability for dynamic flow demands
DevoFlow [17]	NOX, TCAM, OpenFlow	Modifies OpenFlow model by breaking the coupling between network control and global visibility without imposing unnecessary costs	Provision of fine-grained flow management and simplification of OpenFlow switches	Does not reveal how to deploy the default path
Infinite CacheFlow [91]	Ryu Controller, OpenFlow 1.0, Open vSwitch	Proposes a hardware/software hybrid switch design that relies on rule caching to provide large rule tables at low cost	Flow rule dependencies mapped to a graph, flow rule segregation, preserves the network rule semantics	Reactive flow placement Overhead and inconsistent dependencies of flow rules
Switch Reduce [92]	NOX Controller, OpenFlow	Number of rules at any switch should not exceed the set of unique processing actions to decrease switch state	Controller intervention minimization by stateful data plane	Efficient memory utilization and deletion mechanism for useless entries is lacking
Flow Entry MGT Scheme [93]	Mininet, Open vSwitch	Resolves the cache-missing problem and keeps recently used flow entries, which increases flow entry matching ratio	Enhances the flow rule matching in flow tables of OpenFlow-enabled switches	Less efficient in networks where the behaviour and demand are not specified
Traffic Engineering [94]	TCAM, OpenFlow, ITALYNET	Proposes a flow rule multiplexing approach that optimizes flow rule allocation and traffic engineering	Enhances QoS	Considers only identical flow rules that may not be semantically streamlined
DomainFlow [95]	Virtual extensible LAN, TCAM, VMware vCloud	Presents a flow-level control- and granularity-based mechanism in ethernet switches by using OpenFlow protocol	Exact match rule mechanism supports granular security processing	Only supports limited flow rules and fixed number of switches
Source Flow [96]	TCAM, OpenFlow, Open vSwitch	Handles many flows without affecting flow granularity	Offers a comprehensive mechanism for scalability	Synchronization problem
DIFANE [102]	NOX, OpenFlow Switch, TCAM	Load balances the functionalities between network switches and controller	Enhances the SDN scalability and decreases the controller load	Increases load at switches and under-utilizes the controller
Efficient flow rule installation [106,107,109]	Network performance, QoS and security increases	Detects the network reachability change and computes reconfiguration	Supports resiliency	Introduces end-to-end delay due to policy composition and version comparison

**Table 3:** Summary of network security and management.

Studies	Techniques	Description	Strengths	Weaknesses
Advance reservation access control [110]	Ryu controller, OVS switches, ESNet 100 G SDN testbed	Guarantees exclusive access of network resources to a certain flow for which the user/app is authorized	Efficiently protects authorized flows from competing with the network traffic	Need to consider path computation and resource scheduling functions, QoS
Verifying reachability [111]	Z3 version 4.4.2, z Intel Xeon processors with 256 GB of RAM	Verifies reachability by slicing complex networks into small networks according to the network-wide verifications	Provides tool to verify networks in the presence of middleboxes	Middlebox code is not verified, which can affect network traffic by sending packets to invalid interfaces
Systematically troubleshoot networks [112]	OpenFlow, TCAM	Helps network administrators to troubleshoot bugs and their root causes to verify that networks are operating correctly	Provides a useful tool to troubleshoot bugs and their root causes	Does not suggest integrating the program semantics into network troubleshooting tools
SRV [113]	Floodlight, Java, OpenFlow App	Forwards warning messages and refuses the identified flow rule instantly on detection of malicious flow rules	Helpful to detect malicious flow rules	Only discusses priority-based mechanism; other attack scenarios should be analyzed
SDN-Actors [114]	Erlang, Scala, Akka, OpenFlow	Models network applications using actors and verifies various correctness properties via existing model-checking mechanisms	Offers framework to model and verify SDN programs using static and dynamic verification tools to validate network behaviour	Proposed mechanism only tries to uncover programming errors by checking only the SDN program
Reverse update [115]	Python, OpenFlow switch, naive controller	Ensures maintenance of flow rules characteristics during the transition time	Provides a technique to preserve flow rule properties during the network policy change	Lacks an investigation of the effects of network policy change by analyzing packet violations
SVM [116]	Mininet emulator, Floodlight controller	Support vector machine (SVM) algorithm is utilized to judge the traffic and carry out DDoS attack detection	Detects DDoS attacks in SDN to enhance network security	Although this research has the ability to detect DDoS attacks of ICMP traffic, it is less efficient
FUPE [127]	iFogSim, MATLAB R2018a, OpenFlow protocol	Security enhancement against TCP SYN flood attacks in fog nodes via SDN paradigm	Node trust profiling	Fault tolerance not considered
Security architecture [128]	Mininet-WiFi/ethernet, Openday light	Secure and energy-consumption-aware communication in cyber-physical systems (CPS)	Transactional alteration localized using blockchains	No real testbed, only simulation-based evaluation
DHCPguard [131]	Floodlight, ONOS, POX	Sends messages to controller and blocks malicious nodes at forwarding device interfaces	POX controller is designed for DHCP starvation attack mitigation	DHCP failure mitigation not considered
Strengthen SDN security [132]	Mininet, POX controller	Strengthening security assurance via protocol dialect approach	Enterprise security	Scalability limitation
SDN-based edge computing [137]	MATLAB, SDN controller	SDN-supported authentication, routing from end device to edge server, and inter-edge servers’ communication	Lightweight authentication method, activity migration	Single SDN control channel, low performance of edge server with scaled malicious attempts
Near-real-time security [138]	Python, Keras, Mininet, Floodlight, Mininet	Coagulation of the SDN controller using CNN, deployed in any ISP from malicious IoTs	Control channel security	Control channel overhead not discussed
On-the-fly [139]	Floodlight controller, Java, Mininet	Integrates online learning method to limit packet-in rate while tending to the controller queue and switch space capacity	Malicious nodes remain restricted until they are identified as trustworthy	Scalability (hybrid controller placement)
Securing a smart healthcare system [143]	Android, Arduino Nano V3.0 ATMEGA328, Linux, Python	Helpful for patient data preservation and blocking unauthorized access	Provision of healthcare system security	Lacking in integration of SDN control function

**Table 4:** Summary of memory management studies.

Studies	Techniques	Description	Strengths	Weaknesses
Optimal IP routing tables [148]	Internet backbone routing tables	Proposes the ESPRESSO heuristic to minimize the logic to compress the prefix-based match field	Proposed mechanism is helpful for effective utilization of TCAMs	Implemented and tested in traditional IP networking only
Effective switch memory management [151]	Floodlight, Open Daylight, OVS Switch	Based on intelligent flow management strategy to combine adaptive idle timeout values for flow rules and proactive eviction mechanism for TCAM	Beneficial for effective TCAMs utilization	Initial idle timeout, max idle timeout, and rate of timeout increase KPIs and are not considered to gauge efficiency
OpenFlow timeouts demystified [152]	OpenFlow 1.2, CAIDA/32 Dataset, UNIV dataset	Provides hybrid flow table management that combines timeout value with control plane eviction messages	Provides analysis of idle timeout by considering miss rate and flow table size	Dynamic setting of timeout values based on network conditions is missing
OPTree [153]	C++, binary search	Addresses the problem of flow rule placement in firewalls based on ACLs and reduces redundancies	Reduces the number of flow rules during flow rule placement	Lacks a consideration of the network topology change
Flowstat [154]	POX, Mininet	Computation of flow rules for the identified optimal paths and flow rule redistribution	Avoids congestion on network switches	Limited link failure and fault tolerance capability
Lossy compression of packet classifiers [157]	Gigabit ethernet Cisco 6500 switch, WireShark	Offers packet classification approach to find a classifier of optimal size to categorize the network traffic	Classify network traffic for effective TCAM usage of switches	Lacks compressing flow rules to classify a high portion of the traffic
Compressing forwarding tables for data center scalability [159]	TCAM, switches	Each forwarding table column is encoded separately via a dedicated variable-length binary prefix encoding	Offers a useful approach to compress forwarding tables, which is quite helpful in data center virtualization	Can be extended to investigate how other memories (CAM, TCAM) can be utilized to compress forwarding tables

**Table 5:** Summary of SDN simulators and emulators.

Studies	Language	Description	Strengths	Weaknesses
Mininet [164]	Python	Offers a rapid prototyping workflow and virtualization functionalities to assist network developers	The emulation tool, which merges several best features of emulators, hardware testbeds, and simulators	Emulated topology can grow only with residing machine resources
Distributed OF Testbed (DOT) [171]	Java	Supports a cluster of computers that guarantee computation and network resources to switches, hosts, and links	Facilitates large SDN deployments by distributing the workload over a cluster of nodes	Limited number of physical machines to emulate, lacking dynamic scalability and multi-user support
OFNET [172]	Python	Provides built-in functionalities to test and debug, as well as traffic generation and monitoring tools	Helpful in generating network traffic, monitoring of OpenFlow messages and analyzing performance of SDN controller	Needs to be extended for large L2 network and cloud emulation platform
ViNO [173]	Java	Helps to create arbitrary network topologies via Open vSwitches	Domain-specific language for topologies and VM migration in least time	Scalability is not specified
EstiNet [175]	C	Any real application program can run on a simulated host without any modification	Provides accuracy, quickness, repetition, and scalability and supports kernel-reentering simulation methodology	Not scalable to a single process, and results cannot be repeated
FS-SDN [176]	Python	Supports realistic test and validation of standard networks	Scalable and accurate simulation tool	Limited debugging and tracing capabilities
OMNeT++ [178,179]	C++	Used in network modeling, multiprocessors, and different distributed or parallel systems	Popular extensible, modular, component-based scalable simulation tool	Its kernel is in C++ and can only run with modern C++ compiler
NS-3 [181]	C++	Offers help for OpenFlow to program network devices	Can add new protocols, supports the lowering of distance between real network and simulated network	Limited visibility of visual interface for creating topology

**Table 6:** Summary of SDN Programming Languages.

Studies	Programming Paradigm	Description	Strengths	Weaknesses
Frenetic [183,184]	Declarative (functional)	Useful for the categorization and accretion of network traffic	Facilitates modular reasoning	Lack in flow matching and monitoring services
NetCore [187]	Declarative (functional)	It is the successor of Frenetic and carries an enhanced policy management library	Effective handling of controller and switch interaction	Does not support flow matching and monitoring services
Nettle [188]	Declarative (functional, reactive)	Low-level programming language that deals with streams and not with events	Supports dynamic policies, traffic engineering, and load balancing	Does not consider event-driven approach
Procera [189]	Declarative (functional, reactive)	Helps in portraying reactive and temporal behaviors	Good for reactive applications and protocols	Does not support basic flow matching and monitoring
FML [191]	Declarative (data flow, reactive)	High-level language based on non-recursive Datalog [151]	Provides efficient and flexible policies	Limited QoS and monitoring services
Flog [193]	Declarative (logic), event-driven	Event-driven programming that adopted ideas from FML and Frenetic	Supports basic flow matching and monitoring capabilities	Limited security and traffic engineering capabilities
Frenetic OCaml [194]	Declarative (functional)	Utilizes proactive flow rule installation and handles the low-level details of the switch to controller	Effective flow rule installation for efficient communication	Does not support flow matching and monitoring services
Pyretic [195]	Imperative	Helps in specifying static and dynamic forwarding policies to assist in developing network applications	Provides flexible policy making and deployment	Limited flow matching, virtualization, and monitoring capabilities
FlowLog [209,210]	Declarative (functional)	Offers programming for SDN network applications and supports model checking	Provides basic flow matching and monitoring facilities	Does not support traffic engineering and virtualization
FatTire [211]	Declarative (functional)	Used for writing fault-tolerant network applications	Supports basic flow matching and traffic engineering functionalities	Does not provide network monitoring
NetKat [199,202]	Declarative (functional)	Uses Kleene algebra with tests (KAT) [162], based on equational theory, for network programming	Provides sequential and parallel composition capabilities	Does not support external interface monitoring and QoS
Merlin [197,204]	Declarative (logic)	Based on declarative language and useful for distributing and coordinating policy implementation	Supports more secure data processing, flow matching, and monitoring	Does not support link failure and query language
Ponder Flow [198]	Policy specification language	PonderFlow provides mechanisms for implementing access control and network abstractions	Supports dynamic policy language and basic flow matching	Limited monitoring and virtualization capabilities
NOF [207]	Declarative	Enables network application to design the network according to the application requirements	Supports basic flow matching, topology slicing and external interface for monitoring	Limited security and traffic engineering functionalities
Kinetic [208]	Domain-specific language	Helps network operators to control the dynamic state of their network	Inherits runtime features of Pyretic and best flow matching	Limited traffic engineering and monitoring capabilities

**Table 7:** Comparison of SDN controller platforms.

Studies	Techniques	Description	Strengths	Weaknesses
Beacon [212]	Java	Uses ad hoc NBI and SBI with OpenFlow 1.0	Offers high-performance flow processing capabilities	No consistency and limited scalability
Beehive [213]	GO language	Distributed control plane that utilizes REST northbound API and southbound API with OpenFlow specification	Supports multi-threading and good consistency	Weak documentation and reliability
DCFabric [214]	C and JavaScript	Supports Linux platform along with CLI and WebUI.	Supports multi-threading and has a modularity functionality	Limited scalability
Disco [215]	Java	Based on distributed flat architecture that utilizes northbound, southbound, and east/westbound API with REST with OpenFlow 1.0 and AMQP, respectively	Good modularity and strong inter-domain connectivity	Limited documentation and reliability
Faucet [216]	Python	Utilizes SBI with OpenFlow 1.3	Supports multi-threading with good consistency	Limited scalability
Floodlight [217]	Multi-threaded Java	Utilizes REST, Java, RPC, and Quantum northbound API and southbound API with OpenFlow 1.0 and 1.3.	Strong consistency	Very limited scalability and reliability
FlowVisor [218]	C	Provides functions to slice the network resources and is located between guest controllers and switching devices	Good for research experiments and provides slices for several network portions	Limited consistency
HyperFlow [219]	C++	HyperFlow is implemented in C++ and utilizes SBI with OpenFlow 1.0 and east/westbound API with publishing and subscribing messages	Moderate scalability and reliability	Supports proprietary licenses, multi-threading, and no consistency
Kandoo [220]	C, C++, Python	Utilizes Java RPC NBI and SBI with OpenFlow 1.0–1.2	Very good scalability and utilizes Linux supporting platform and proprietary license.	Limited reliability
Loom [221]	Erlang	Provides an experimental network switch controller that implements the OpenFlow 1.3.x and 1.4 protocols	Offers scalability and robustness for large-scale implementations	Limited consistency
Maestro [222]	Multi-threaded Java	Exploits parallelism along with additional throughput optimization techniques	Supports multi-threading and has a fair modularity	No consistency or reliability
MsNettle [223]	Multi-threaded Haskell	Utilizes SBI with OpenFlow 1.0 and Linux platform in CLI mode	Supports proprietary licenses and has a good modularity.	No consistency and limited documentation
Meridian [224]	Java	Utilizes REST northbound API and southbound API with OpenFlow 1.0 and 1.3	Cloud-based platform that supports multi-threading	No consistency and reliability
Microflow [225]	C	Utilizes Socket NBI and SBI with OpenFlow 1.0–1.5 and uses Linux platform with CLI and WebUI modes	Supports multi-threading and has good scalability	No consistency and reliability
NODE FLOW [226]	Java Script	Utilizes JSON NBI and SBI with OpenFlow 1.0	Good reliability	Cisco license and limited documentation
NOX [227]	C++	Utilizes ad hoc NBI and SBI with OpenFlow 1.0 and supports Linux platform in CLI and WebUI modes	Supports GPL 3.0 licenses and multi-threading (NOX-MT).	Low modularity with no consistency
ONIX [228]	C++	It utilizes Onix API, NBI, and SBI with OpenFlow 1.0, OVSDB, and east/westbound API with Zookeeper	Supports multi-threading and has a good modularity	Supports proprietary licenses and weak consistency
ONOS [229]	Java	Utilizes REST and Neutron NBI and SBI with OpenFlow 1.0 and 1.3 and east/westbound API with Raft	Supports Apache 2.0 licenses and multi-threading functionality	Weak consistency in cases
Open Contrail [230]	C, C++, Python	Utilizes REST NBI and SBI with BGP and XMPP and supports Linux platform with CLI and WebUI modes	Supports Apache 2.0 and multi-threading functionality and high modularity	Limited scalability
Open Daylight [231]	Java	Utilizes REST, RESTCONF, XMPP, and NETCONF NBI and SBI with OpenFlow 1.0 and 1.3	Good scalability and reliability as well as strong consistency	Based on Cisco’s ONE SDN controller
OpenIRIS [232]	Java	Utilizes REST NBI, SBI with OpenFlow 1.0–1.3, and east/westbound API with custom protocol	Good reliability and provides multi-threading	Weak consistency and no support for Openstack
OpenMul [233]	C	Utilizes REST NBI and SBI with OpenFlow 1.0, 1.3, OVSDB, and Netconf	Supports Linux platform in CLI mode and good reliability	Weak scalability and consistency
PANE [234]	Haskell	Utilizes PANE NBI, SBI with OpenFlow 1.0 and Zookeeper east/westbound API	Supports BSD 3.0 licenses and has a fair modularity	Limited reliability with no consistency
POF Controller [235]	Java	Utilizes SBI with OpenFlow 1.0 and POF-FIS and supports Linux platform along with CLI and WebUI	Reliable and scalable	Limited documentation and consistency
POX [236]	Python	Utilizes ad hoc NBI and SBI with OpenFlow 1.0; uses Linux, MacOS, and Windows platform; and supports CLI and GUI	Consistent controller platform	Limited reliability and scalability
Ravel [237]	Python	Utilizes ad hoc NBI and SBI with OpenFlow 1.0 and supports Linux platform along CLI and WebUI, with fair documentation	Good reliability with strong consistency	Very limited scalability
Rosemary [238]	C	Utilizes ad hoc NBI and SBI with OpenFlow 1.0, 1.3, and XMPP and supports Linux platform, along with CLI and WebUI	Supports Proprietary and multi-threading functionality with good modularity	No scalability and consistency
Ryu [240]	Python	Utilizes REST NBI and SBI with OpenFlow 1.0–1.5 and supports Linux and MacOS platforms with CLI mode	Good modularity and support for OpenStack	Limited scalability and weak consistency
SMaRt Light [241]	Java	Utilizes REST NBI, SBI with OpenFlow 1.3, and east/westbound API	Supports Linux platform in CLI mode and has good reliability and consistency	Proprietary license and limited scalability
TinySDN [242]	C	Utilizes SBI with OpenFlow 1.0 and supports Linux platform with CLI mode	Supports BSD 3.0 licenses and modularity	No multi-threading functionality or consistency
Trema [243]	C, Ruby	Utilizes ad hoc NBI and SBI with OpenFlow 1.0 and supports Linux platform in CLI mode	Supports GPL 2.0 licenses and has good modularity	No consistency and reliability
Yanc [244]	C, C++	Utilizes REST NBI and SBI with OpenFlow 1.0–1.3 and supports CLI mode	Provides reliable communication	Limited documentation and no consistency
ZeroSDN [245]	C++	Uses REST NBI and SBI with OpenFlow 1.0 and 1.3 and supports Linux platform with CLI and WebUI modes	High modularity and consistency	Does not support multi-threading and not scalable

**Table 8:** Comparison of existing papers in SDN.

Studies	Year	Area Discussed	Methods Used
Akyildiz et al. [247]	2014	Flow management, fault tolerance, topology update.	Supervised and unsupervised learning for traffic engineering
Xie et al. [248]	2017	Routing optimization, QoS, resource management security	Supervised and unsupervised learning for traffic engineering
Matlou et al. [249]	2017	SDN, wireless sensor networks	Supervised, reinforcement, and unsupervised learning
Jose et al. [250]	2017	Traffic classification and security	Comprehensive study on security and traffic using traditional and formal methods
Sultana et al. [251]	2018	Intrusion detection	Deep and unsupervised learning for IDS
Cui et al. [252]	2018	Traffic profiling	Different supervised and unsupervised learning methods
Loung et al. [253]	2018	Network function virtualization and QoS	Using machine learning and mathematical methods
Kreutz et al. [254]	2014	Comprehensive survey on SDN core concepts	General discussion based on the traditional and Openflow concepts
Wang et al. [255]	2015	Heterogeneous networks	AI-based techniques
Jamshidi et al. [256]	2016	Cybersecurity intrusion detection	Supervised learning and unsupervised learning
Mohammed at al. [257]	2019	Traffic classification and prediction	Supervised, reinforcement, and unsupervised learning
Tam et al. [258]	2018	Security in network	Machine and deep learning for security
Amin et al. [259]	2018	General concepts of hybrid SDN and terminologies	Categorization of hybrid SDN based on different technical models
Priyadarsini et al. [260]	2021	A comprehensive state-of-the-art progress report on specific topics of SDN	Classifications of traffic management, load balance, network safety, and controller placement

## Data Availability

Not applicable.
